# Effects of weightlessness on the cardiovascular system: a systematic review and meta-analysis

**DOI:** 10.3389/fphys.2024.1438089

**Published:** 2024-07-26

**Authors:** Rafaella Mendes Zambetta, Étore De Favari Signini, Gabriela Nagai Ocamoto, Aparecida Maria Catai, Nicoly Ribeiro Uliam, Emiliano Santarnecchi, Thiago Luiz Russo

**Affiliations:** ^1^ Physical Therapy Department, Federal University of São Carlos, UFSCar, São Carlos, SP, Brazil; ^2^ Brain4care Inc., São Carlos, SP, Brazil; ^3^ Massachusetts General Hospital, Harvard Medical School, Boston, MA, United States

**Keywords:** cardiovascular system, fluid shift, hemodynamics, spaceflight, weightlessness conditions, ground-based simulations

## Abstract

**Background:** The microgravity environment has a direct impact on the cardiovascular system due to the fluid shift and weightlessness that results in cardiac dysfunction, vascular remodeling, and altered Cardiovascular autonomic modulation (CAM), deconditioning and poor performance on space activities, ultimately endangering the health of astronauts.

**Objective:** This study aimed to identify the acute and chronic effects of microgravity and Earth analogues on cardiovascular anatomy and function and CAM.

**Methods:** CINAHL, Cochrane Library, Scopus, Science Direct, PubMed, and Web of Science databases were searched. Outcomes were grouped into cardiovascular anatomic, functional, and autonomic alterations, and vascular remodeling. Studies were categorized as Spaceflight (SF), Chronic Simulation (CS), or Acute Simulation (AS) based on the weightlessness conditions. Meta-analysis was performed for the most frequent outcomes. Weightlessness and control groups were compared.

**Results:** 62 articles were included with a total of 963 participants involved. The meta-analysis showed that heart rate increased in SF [Mean difference (MD) = 3.44; *p* = 0.01] and in CS (MD = 4.98; *p* < 0.0001), whereas cardiac output and stroke volume decreased in CS (MD = −0.49; *p* = 0.03; and MD = −12.95; *p* < 0.0001, respectively), and systolic arterial pressure decreased in AS (MD = -5.20; *p* = 0.03). According to the qualitative synthesis, jugular vein cross-sectional area (CSA) and volume were greater in all conditions, and SF had increased carotid artery CSA. Heart rate variability and baroreflex sensitivity, in general, decreased in SF and CS, whereas both increased in AS.

**Conclusion:** This review indicates that weightlessness impairs the health of astronauts during and after spaceflight, similarly to the effects of aging and immobility, potentially increasing the risk of cardiovascular diseases.

**Systematic Review Registration:** https://www.crd.york.ac.uk/prospero/, identifier CRD42020215515.

## Background

Interplanetary travel has become a significant objective for humanity, but it presents an enormous challenge: protecting the human body. Astronauts are exposed to several stressful situations, including cosmic radiation, sleep deprivation, low levels of physical activity, and microgravity (Hughson et al., 2018). The human body, especially the cardiovascular system, relies on the Earth’s gravity for proper function. Microgravity exposure leads to substantial changes in this system, which can endure for months after astronauts return to the ground (Demontis et al., 2017; Hughson et al., 2018).

Spaceflight experiments are rare and costly; hence, investigators seek to comprehend the impacts of microgravity on the cardiovascular system adaptation by implementing ground models including head-down tilt bed rest and immersion (Oluwafemi and Neduncheran, 2022). On Earth, there is a hydrostatic gradient, which is implicated in fluid pressures according to gravity. For example, the pressure is higher in the lower limbs than in the intracranial cavity. The lack of gravity in space leads to a fluid shift in the hydrostatic gradient, resulting in a uniform pressure of fluid throughout the body, which causes fluids to move upwards (Demontis et al., 2017; Hughson et al., 2018).

Astronauts may experience symptoms associated with the fluid shift, such as puffy face, swollen jugular veins, heart chamber distension, headaches and head “stuffing,” flu-like symptoms, and bird legs (thinner lower limbs), due to a reduction of around 10% in the blood volume of the lower limbs (Norsk, 2020). The literature suggests that long-duration space missions lead to an increase in venous return and cardiac preload (Fortrat et al., 2017), resulting in an increase in cardiac output (Norsk et al., 2015; Hughson et al., 2016; Marshall-Goebel et al., 2019). Cardiovascular dynamics changes may contribute to cardiac atrophy, as a cardiac muscle adaptation (Aubert et al., 2005; Shen and Frishman, 2019). In this sense, microgravity effects in heart rate (HR), blood pressure (BP) and stroke volume (SV) responses are still discussed. Some authors suggest that HR and SV tend to increase (Norsk et al., 2015; Mulavara et al., 2018; Marshall-Goebel et al., 2019; Wood et al., 2019), while systolic arterial pressure (SAP) decreases (Hughson et al., 2016; Fu et al., 2019), as a potential consequence of cardiac adaptation.

Arterial morphology and function can also be modified by microgravity, especially in the intracranial cavity (Arbeille et al., 2016; Lee et al., 2020). The mechanical stress in these arteries causes an enlargement of the artery circumference and thickness of tunica intima and media (Kotovskaya and Fomina, 2010; Grigoriev et al., 2011; Arbeille et al., 2017a). Additionally, weightlessness causes plasmatic hypovolemia and dehydration, leading to a compensatory increase in the sympathetic component to maintain stable cardiac output (Di Rienzo et al., 2008; Jimenez et al., 2012). Chronically, peripheral baroreceptors tend to accommodate, impairing baroreflex sensitivity (BRS) (Di Rienzo et al., 2008). Studies propose that the sympathetic component of Cardiovascular autonomic modulation (CAM) is predominant in both spaceflights and Earth simulation experiments, with reduction of heart rate variability (HRV) and baroreflex sensitivity (BRS) (Verheyden et al., 1985; Arzeno et al., 2013; Xu et al., 2013; Rusanov et al., 2020). On the contrary, Vandeput and colleagues (Vandeput et al., 2013) documented a reduction in both the sympathetic and parasympathetic components of HRV following prolonged spaceflight, indicating distinct delayed adaptation to microgravity. As a result, the available evidence on CAM regulation is not entirely conclusive.

Finally, the aforementioned cardiovascular alterations lead to orthostatic intolerance, resulting in inadequate cerebral perfusion and deconditioning in astronauts (Shen and Frishman, 2019; Gallo et al., 2020). This situation raises concern in circumstances that require precise performance, like in exploratory missions, emergencies, or extravehicular activities. Moreover, astronauts may experience health implications, including visual impairment and intracranial pressure syndrome (Platts et al., 2014), an increased risk of arrhythmia and cardiovascular diseases, even upon returning to Earth (Gallo et al., 2020). Therefore, this meta-analysis and systematic review seeks to identify the acute and chronic effects of microgravity (or corresponding analog models on Earth) on the anatomy and function of the cardiovascular system.

## Methods

This systematic review followed the Preferred Reporting Items for Systematic Reviews and Meta-Analysis (PRISMA) guidelines (Page et al., 2021). To systematize and simplify the search and data collection process, we utilized the State of the Art through Systematic Review (StArt) tool (available at http://lapes.dc.ufscar.br/tools/start_tool). Furthermore, we registered this systematic review in the International Prospective Register of Systematic Reviews (PROSPERO) with the identifier CRD42020215515.

## Data sources and search strategy

Literature searches were conducted utilizing the subsequent databases: CINAHL with Full Text (EBSCO), Cochrane Library, Scopus, Science Direct, PubMed (via National Library of Medicine) and Web of Science (Thomson Scientific/ISI Web Services). MeSH terms were utilized to identify relevant keywords, and the resulting string of terms was subsequently employed. During the searched period from January 2013 to 2023, database searches were conducted for publications in English language containing the following keywords: “humans'' OR “astronauts” AND “weightlessness” OR “weightlessness Simulation” OR “space flight” AND “cardiovascular system” OR “hemodynamics” OR “fluid shifts.” An update was made in October 2023. Supplementary Table S1 illustrates all the applied filters, combinations, and searches.

## Eligibility criteria

The main question of this study was based on the PICOS structure (P: population; I: intervention; C: comparison; O: outcomes; and S: study design). The subjects of interest were astronauts and healthy adult humans. Three weightlessness simulation experiments—Head-Down Tilt (HDT), Dry Immersion (DI) and Parabolic Flight (PF)—were selected due to the presence of fluid shift in each model, albeit at varying magnitudes. They are suitable models for investigating the physiological alterations resulting from weightlessness, particularly concerning the cardiovascular system (Norsk et al., 2015; Ploutz-Snyder, 2016). Comparisons using control groups or pre-post interventions were assessed. The cardiovascular outcomes considered were grouped into functional or anatomical modifications. Only quantitative studies were accepted.

Accordingly, the inclusion criteria were healthy adult humans and weightlessness effects on the cardiovascular system. We excluded studies with animal models, children, elderly population, and diseases; studies focusing solely in hypergravity or hypogravity, such as Martian and lunar gravity, and not microgravity; and qualitative studies, narrative reviews, meta-analysis, and systematic reviews, gray literature (e.g., governmental reports, theses, etc.), theoretical and opinion articles, conference abstracts, personal blogs, and social media were excluded.

## Selection process

After removing duplicates, two reviewers (RMZ and EFS) independently selected articles that met the eligibility criteria based on their titles and abstracts. The selected articles were then retrieved for full-text reading to confirm that they met all inclusion criteria. Any disagreements between the two reviewers were resolved by consulting a third reviewer (GNO) to determine whether the study was eligible.

## Data analysis

### Data extraction and synthesis

We divided the studies into three categories: spaceflight (SF), chronic simulations (CS), and acute simulations (AS) (Tables 1–3). No specific timeframe was defined for spaceflight and weightlessness simulations. Studies with a duration exceeding 24 h were classified as CS due to the involvement of later blood pressure regulation mechanisms, including renal ones (Hall and Guyton, 2011). Studies with a duration of less than 24 h (<24 h) were categorized as AS.

**TABLE 1:** Summary of main findings in spaceflight studies.

Spaceflight					
Authors, year published	Participants	Study design	Cardiovascular variables evaluated	Tools	Main outcomes
1. Arbeille et al. (2015)	10 astronauts (7 males and 3 females; age 47 ± 5 years; height 172 ± 8 cm; weight 69 ± 12 kg)	Longitudinal study. Astronauts spent 4–5.5 months aboard the ISS. Measurements: before flight, inflight (on day 15 and between day 115 and 165), and 4 days postflight	JV, PV, FV, GastV, TibV CSA; JV and PV volume; JV volume/PV volume ratio	Echography	JV and FV CSA increased early and late inflight, along with PV and JV volume, but in different magnitudes, resulting in a higher JV/PV ratio. TibV and GastV CSA decreased. TibV CSA remained reduced postflight
2. Arbeille et al. (2016)	10 astronauts (7 males and 3 females; age 47 ± 5 years; height 172 ± 8 cm; weight 69 ± 12 kg)	Longitudinal study. Astronauts spent 4–5.5 months aboard the ISS. Measurements: before flight, inflight (on day 15 and between day 115 and 165), and 4 days postflight	CA and FA IMT, CSA and diameter	Echography	Both CA and FA IMT increased early and late in spaceflight. CA IMT remained increased postflight
3. Arbeille et al. (2017)	10 astronauts (7 males and 3 females; age 47 ± 5 years; height 172 ± 8 cm; weight 69 ± 12 kg)	Longitudinal study. Astronauts spent 4–5.5 months aboard the ISS. Measurements: before flight, inflight (on day 15 and between day 115 and 165), and 4 days postflight	SAP and DAP; CA and FA IMT, strain; elastic modulus; stiffness index and distensibility	Echography and Doppler ultrasonography	DAP reduced late and after spaceflight. CA stiffness indices had a reduction for stain and distensibility, while elastic modulus and stiffness index increased late inflight. CA and FA IMT were elevated inflight
4. Arbeille et al. (2021)	14 astronauts (11 males and 3 females; age 47 ± 6 years; BMI 26.4 ± 3 kg/m²	Longitudinal study. Astronauts spent 210 ± 76 days aboard the ISS. Measurements: before flight, inflight (on day 45 and day 150), and 40 days and 180 days postflight	JV volume; PV CSA; MCV velocity	Doppler ultrasonography	JV volume increased inflight. PV CSA and MCV velocity were also elevated inflight, but more pronounced at day 150
5. Blaber et al. (2022)	27 astronauts (20 males and 7 females; age 39 ± 5 years)	Longitudinal study. Astronauts spent 8–16 days on shuttle missions. Measurements were made for 5–10 min during a supine-to-stand test (STS) before flight and 1–2 h after landing and 3 days postflight	BRS gain and FTA (HF and LF), and causality between SAP and RR (SAP→RR: neural reflex, and RR→SAP: mechanical coupling)	ECG and digital infrared photoplethysmography	FTA (HF) and neural reflex causality reduced after flight
6. Fortrat et al. (2017)	24 male astronauts (age 44.3 ± 6.1 years; height 177 ± 5 cm; weight 82.6 ± 6.7 kg; BMI 26.4 ± 2.3 kg/m²)	Longitudinal study. Astronauts spent between 124 and 192 days aboard the ISS. Measurements: before flight; inflight (once before and once after 3 months in space); and postflight (on the landing day and 8 days after)	Calf volume; venous filling function; venous filling index; venous emptying	Occlusive air plethysmography	Calf volume reduced during flight. Venous filling function and index increased early inflight. Venous emptying diminished early and late inflight
7. Fu et al. (2019)	12 astronauts (8 males and 4 females; age 48 ± 5 years; height 173 ± 8 cm; weight 72 ± 13 kg)	Longitudinal study. Astronauts spent about 6 months aboard the ISS. Measurements: before flight, inflight (at days 5, 30, 75 and 15 days before return to Earth), and on the landing day	HR; SAP, DAP and MAP; PP; BPV; skewness and kurtosis of SAP.	Digital infrared photoplethysmography	SAP, MAP and PP decreased in space. SAP skewness and kurtosis increased inflight. HR increased only on postflight
8. Hoffmann et al. (2019)	8 cosmonauts (7 males and 1 female; age 46.5 ± 5.3 years; height 176 ± 6.2 cm; weight 77.6 ± 8.2 kg)	Longitudinal study. Astronauts spent about 6 months aboard the ISS. Measurements: before flight and twice postflight (on day 4 and 8 after landing)	HR; peripheral and estimated central SAP and DAP; aortic PWV; systolic and PP amplification	Blood pressure oscillometry	HR increased postflight. Systolic amplification was lower at day 4 postflight compared to baseline
9. Hughson et al. (2016)	9 astronauts (5 males and 4 females; age 40–56 years; height 174 ± 7 cm; weight 69 ± 15 kg)	Before-and-after. Astronauts spent between 146 and 193 days aboard the ISS. Measurements: before flight and once postflight (22–38 h after landing)	HR; SAP, DAP and MAP; SV; CO; carotid PP, CA stiffness and distensibility coefficient; posterior tibial artery PP; PWTT.	ECG, digital infrared photoplethysmography, carotid applanation tonometry, ultrasonography	HR increased postflight. CA stiffness increased and distensibility reduced. PWTT was faster postflight
10. Ishihara et al. (2020)	5 male astronauts	Longitudinal study. Astronauts spent between 115 and 199 days aboard the ISS. Measurements: before flight and four times after flight (on the landing day, after 7 days, 1 month and 3 months postflight)	Blood flow (on the central region of the gastrocnemius muscle)	Laser blood flowmetry	Blood flow reduced at days 1 and 7 postflight and had recovered to baseline levels 1 month after landing
11. Iwasaki et al. (2021)	11 astronauts (10 males and 1 female; age 46.0 ± 7.0 years; height 178.7 ± 6.1 cm; weight 82.5 ± 8.5 kg)	Longitudinal study. Astronauts spent 4–6 months on the mission. Measurements: preflight and twice postflight (0–3 days and 138 ± 62 days after landing)	MCA velocity; MAP; and HR.	Doppler ultrasonography, echocardiography, tonometry and sphygmomanometry	HR and MCA velocity showed a significant increase early postflight
12. Khine et al. (2018)	13 astronauts (9 males and 4 females; age 49 years)	Longitudinal study. Astronauts spent about 6 months aboard the ISS. Measurements: MRI–preflight, within 5 days postflight and 3–8 weeks postflight. Holter monitoring–preflight; inflight on days 14, 30, 75, 135, and 15 days before landing; and on the landing day	LA volume; RA area, longitudinal and transversal diameter; atrioventricular plane displacement; P-wave amplitude and duration	Cardiac MRI and continuous ECG (12-lead Holter 48 h)	LA volume transitorily increased postflight. P-wave amplitude showed a decrease duringflight and on landing day in some ECG derivations. One astronaut had a large increase in supraventricular ectopic beats, but not atrial fibrillation
13. Lee et al. (2015)	20 astronauts (17 males and 3 females; age 46 ± 5 years; height 175 ± 7 cm; weight 81 ± 9 kg) and 65 Shuttle astronauts (50 males and 15 females; age 43 ± 7 years; height 174 ± 7 cm; weight 78 ± 13 kg)	Longitudinal study. 20 astronauts spent 177 ± 21 days aboard the ISS and 65 astronauts spent 12 ± 3 days at space shuttle missions. Measurements: orthostatic tolerance test was done before flight, on the landing day or 1 day after, 3 and 10 days after landing	HR; SAP, DAP and MAP; SV; CO; SVR; cardiovascular variability index	ECG, digital infrared photoplethysmography, 2D-echocardiography and Doppler ultrasonography	HR was elevated on the landing day. BP in general tended to be elevated postflight. SV and CO were lower for ISS group on the landing day. Cardiovascular variability index increased in both groups, but it showed higher values for ISS astronauts
14. Lee et al. (2020)	13 astronauts (10 males and 3 females; age 46 ± 8 years; height 176 ± 7 cm; weight 79 ± 11.3 kg; BMI 25.5 ± 2.6 kg/m²)	Longitudinal study. Astronauts spent 189 ± 61 days aboard the ISS. Measurements: preflight, inflight (on days 15, 60 and 160) and postflight 5 days after landing	CA IMT and stiffness; global arterial compliance; HR; SAP, DAP and MAP; PP; SV; CO; SVR	Doppler ultrasonography	CA diameter increased inflight, SV and global artery compliance reduced during flight. HR, CO and SVR were elevated postflight
15. Liu et al. (2015a)	3 astronauts (1 female and 2 males; age 33–49 years)	Longitudinal study. Astronauts spent about 15 days on space mission. Measurements: before flight, inflight (3–6 days after launch) and postflight (4–6 days after landing)	Average HR, amplitude, maximum and minimum values of HR	ECG	HR had elevated values after flight. HR amplitudes had an important decrease inflight. Maximum HR values increased during and after flight. Minimum HR was elevated postflight
16. Marshall-Goebel et al. (2019)	11 astronauts (9 males and 2 females; age 46.6 ± 6.3 years; BMI 26.4 ± 3 kg/m²)	Longitudinal study. Astronauts spent 210 ± 76 days aboard the ISS. Measurements: before flight, twice inflight (at day 50 and day 150), and once after flight	SV; CO; HR; SAP, DAP and MAP; JV pressure, CSA and blood flow	Blood pressure oscillometry, vein press compression sonography and Doppler ultrasonography	SV and CO, along with JV CSA increased inflight. JV blood flow demonstrated stagnant flow or even retrograde flow pattern in some astronauts. Two subjects presented an occlusive thrombus
17. Mulavara et al. (2018)	13 astronauts (11 males and 2 females; age 47 ± 5 years; height 178 ± 6 cm; weight 84 ± 14 kg)	Controlled clinical trial. Astronauts spent 159 ± 17 days aboard the ISS. Measurements: before flight and three times postflight (1 day, 8 days and 37 days after landing)	HR and MAP	ECG and digital infrared photoplethysmography	HR significantly increased postflight
18. Norsk et al. (2015)	8 male astronauts (age 45–53 years; height 169–188 cm; weight 70–99 kg; BMI 22.5–28.6 kg/m²)	Longitudinal study. Astronauts spent about 6 months aboard the ISS. Measurements: before flight; inflight (at the days 85 and 192), and >2 months postflight	SAP, DAP and MAP; HR; CO; SVR; and SV	Blood pressure oscillometry and gas rebreathing technique	SAP, DAP and MAP were reduced inflight, while CO and SV were elevated. SVR also decreased in space
19. Otsuka et al. (2015)	7 astronauts (5 males and 2 females; age 52.0 ± 4.2 years)	Longitudinal study. Astronauts spent 172.6 ± 14.6 days aboard the ISS. Measurements: before flight; inflight (at the day 24 ± 5, day 73 ± 5 after launch and 15 ± 5 days before return to Earth); and 36 and 100 days postflight	HRV: HF, LF, VLF and ULF, slope of fractal scaling	Continuous ECG (two-channel Holter 24 h)	Circadian rhythm showed to be present in space. ULF decreased inflight. Slope was less negative in space than on Earth
20. Otsuka et al. (2016)	10 astronauts (8 males and 2 females; age 49.1 ± 4.2 years)	Longitudinal study. Astronauts spent 171.8 ± 14.4 days aboard the ISS. Measurements: before flight; inflight (at days 20.8 ± 2.9, 72.5 ± 3.9 after launch and 19.1 ± 4.1 days before return); and postflight at day 77.2 ± 14.4. Astronauts were divided in two groups: Group 1: decreased HR (n = 7) and Group 2: increased HR (n = 3)	HR; HRV: HF, LF, VLF and ULF, slope of fractal scaling; NN intervals	Continuous ECG (two-channel Holter 24 h)	Considering all 10 astronauts, in general HR decreased in space. NN-intervals were lower in Group 1 than in Group 2. Group 1 tended to respond to space by increasing the average NN interval (decreasing HR). In both groups during flight, ULF decreased, total spectrum power increased and slope was less negative
21. Otsuka et al. (2022)	1 astronaut	Longitudinal study. Measurements: once preflight, twice inflight (at days 18–19 and 326–327), and 103–104 days postflight	HR; HRV LF, HF, VLF, ULF, LF/HF ratio, NN intervals, RMSSD, pNN50, and slope of fractal scaling	Continuous ECG (two-channel Holter 48 h)	HR increased postflight. NN intervals, pNN50 and VLF decreased postflight. Both LF and HF were elevated late inflight, and HF reduced postflight to lower values than baseline. RMSSD significantly decreased only early inflight and postflight. Slope decreased during flight
22. Pavela et al. (2022)	11 astronauts (6 males and 5 females)	Longitudinal study. Measurements: preflight, three times inflight (at days 27, 80 and 168) and 3 days postflight	JV CSA and peak velocity; right-to-left JV CSA ratio; grade of spontaneous echo contrast in JV.	Doppler ultrasonography	Three astronauts had isolated cases of retrograde flow, but none had deep vein thrombosis. Six astronauts presented mild-moderate echogenicity on in the JV. Peak velocity reduced and CSA increased inflight
23. Vandeput et al. (2013)	8 male astronauts (age 43.2 ± 4.4 years; height 179 ± 4 cm; weight 76.0 ± 12.4 kg; BMI 23.7 ± 3.7 kg/m²)	Longitudinal study. Five astronauts spent about 6 months in space and the other 3 for about 10 days. Measurements: before flight, early postflight (5 days after landing) and late postflight (1 month after landing)	HRV: RR interval, SDNN, RMSSD; pNN50; LF and HF powers; LF/HF; and HRV complexity (fractal dimension, detrend fluctuation analyzes; sample entropy; correlation dimension; Lyapunov exponent)	Continuous ECG (Holter 24 h)	HF increased at night and LF/HF ratio decreased. Total spectral power, HF power, RMSSD and pNN50 decreased at postflight. Sympathovagal balance strongly increased early after flight. There was a significant decrease in the complexity variables early postflight
24. Wood et al. (2019)	9 male astronauts (age 45 ± 7 years)	Before-and-after study. Astronauts spent 165 ± 13 days aboard the ISS. Measurements: before flight and within 36 h after return to Earth	SAP and DAP; HR; peripheral artery pulse arrival time (PAT) and its inverse (1/PAT)	ECG and digital infrared photoplethysmography	HR and DAP increased after flight
25. Xu et al. (2013)	7 astronauts (1 female and 6 males; age 47 ± 4.6 years; height 176 ± 5 cm; weight 81 ± 9.6 kg)	Longitudinal study. Astronauts spent 144 ± 49 days aboard the ISS. Measurements: preflight, inflight (2–3 weeks after launch and 2–3 weeks before landing), and 1–3 days postflight	HR; HRV: SDRR, SDARR, RMSSD, HF, LF, VLF and ULF powers and normalized units; LF/HF ratio; sample entropy	Continuous ECG (12-lead Holter 24 h)	HR was elevated after flight. HRV reduced in time domain indices (SDRR, SDARR and RMSSD), and in frequency domain indices (LF, HF, VLF and ULF)
26. Yamamoto et al. (2015)	7 astronauts (5 males and 2 females; age 52.0 ± 4.2 years)	Longitudinal study. Astronauts spent 172.6 ± 14.6 days aboard the ISS. Measurements: before flight; inflight (at the day 24 ± 5, day 73 ± 5 after launch and 15 ± 5 days before return to Earth); and 36 and 100 days postflight	HRV: CVRR, SDNN, SDRR, SDARR, RMSSD, HF, LF, VLF and ULF powers, and LF/HF ratio	Continuous ECG (two-channel Holter 24 h)	Both power spectrum and normalized spectrum power for LF, HF, VLF and ULF as well as SDRR, SDARR and RMSSD reduced inflight. Postflight, HF component remained lower

Abbreviations: BMI, body mass index; BPV, blood pressure variability; BRS, baroreflex sensitivity; CA, carotid artery; CO, cardiac output; CSA, cross-sectional area; CVRR, coefficient of variation of RR, intervals; DAP, diastolic arterial pressure; ECG, electrocardiography; FA, femoral artery; FTA, fraction time active; GastV, gastrocnemius vein; HF, high frequency; HR, heart rate; HRV, heart rate variability; IMT, intima-media thickness; ISS, international space station; JV, jugular vein; LA, left atrium; LF, low frequency; LF/HF, ratio, ratio of low frequency and high frequency of heart rate variability; MAP, mean arterial pressure; MCA, middle cerebral artery; MCV, middle cerebral vein; MRI, magnetic resonance imaging; NN, intervals, normalized time between two successive R-waves of the QRS, signal on the electrocardiogram; pNN50, percentage of adjacent NN (R-R) intervals that differ from each other by more than 50 ms; PP, pulse pressure; PV, portal vein; PWTT, pulse wave transit time; PWV, pulse wave velocity; RA, right atrium; RMSSD, root mean square of successive differences between normal heartbeats; RR, interval, time elapsed between two successive R-waves of the QRS, signal on the electrocardiogram; SAP, systolic arterial pressure; SDARR, standard deviation of the set of averaged RR, intervals; SDNN, standard deviation of the interbeat intervals for all sinus beats; SDRR, standard deviation of the R-R intervals; SV, stroke volume; SVR, systemic vascular resistance; TibV, tibial vein; ULV, ultra-low frequency; VLF, very low frequency. (mean ± standard deviation). [minimal–maximal].

**TABLE 2:** Summary of main findings in chronic simulation studies.

Chronic simulation					
Authors, year published	Participants	Study design	Cardiovascular variables evaluated	Tools	Main outcomes
1. Adami et al. (2013)	9 healthy men (age 23 ± 2.5 years; height 179.7 ± 7.3 cm; weight 72.1 ± 9.2 kg)	Longitudinal study. Participants past 35 days in −6° HDT bed rest. Subjects were divided into two groups: tolerant and intolerant to orthostatic posture. Measurements: at the end of HDT and at recovery	HR; MAP; SV; CO; and SVR.	ECG and digital infrared photoplethysmography	Tolerant group: SV, MAP and SVR tended to decrease with the standing position and HR increase. Intolerant group: CO, MAP and SV severe dropped and HR increased. SVR had high values at the interruption of the standing test
2. Arzeno et al. (2013)	20 men (age 32.5 ± 1.3 years; height 175.8 ± 1.8 cm; weight 82.2 ± 2.6 kg) and 10 women (age 37.2 ± 2.8 years; height 160.2 ± 1.7 cm; weight 60.6 ± 3.7 kg)	Longitudinal study. Participants past 60 days in −6° HDT bed rest. Measurements: before protocol, and on days 30 and 60 of HDT.	SAP and DAP; BRS; RR intervals; HRV and BPV (LF and HF)	ECG and digital infrared photoplethysmography	RR interval, BRS, SAP and DAP decreased with bed rest. HRV showed alterations during the entire protocol, in which HF diminished and LF increased
3. Blaber et al. (2022)	19 health man (age 35.0 ± 1.7 years; height 176.0 ± 1.0 cm; weight 72.9 ± 1.7 kg; BMI 23.7 ± 0.4 kg/m²	Longitudinal study. Participants spent 60 days in −6° HDT. Measurements were made for 5–10 min during a supine-to-stand test before HDT, on the last day of HDT and 8 days after	FTA, BRS gain, and causality between SAP and RR (SAP→RR: neural reflex, and RR→SAP: mechanical coupling)	ECG and digital infrared photoplethysmography	Neural reflex (SAP→RR) reduced after HDT.
4. Caiani et al. (2013)	22 healthy men (age 31 ± 6 years)	Longitudinal study. Participants past 5 days in −6° HDT bed rest. Measurements: before protocol, on the last day and 5 days after	RR intervals; QRS-T angle; ventricular gradient magnitude; RTapex; RTend; Tapex; Tarea	Continuous ECG (12-lead Holter 24 h)	Rtapex and Rtend decreased at HDT. Tapex and Tarea decreased at HDT and Tapex increased post-HDT. Ventricular gradient magnitude reduced, and QRS-T angle increased at HDT. Ventricular gradient magnitude reduced after HDT
5. Liang et al. (2014)	8 healthy men (age 26.2 ± 4.1 years; height 171.8 ± 3.0 cm; weight, 63.6 ± 6.2 kg)	Longitudinal study. Participants underwent 9 ambulatory days before bed rest, 45 days of −6° HDT bed rest and 14 ambulatory days after bed rest. Data was collected during all the protocol	HR and HRV (LF and HF)	ECG	HR decreased only at the beginning of HDT. Both LF and HF power decreased in recovery phase
6. Linnarsson et al. (2014)	11 healthy men (age 34 ± 6 years; height 179 ± 7 cm; weight 76 ± 6 kg)	Controlled crossover trial. Participants performed 5 days of −6° HDT. Measurements: before and after HDT	HR; SAP and DAP	ECG and digital infrared photoplethysmography	HR decreased during HDT and increased at recovery phase. Before bed rest, SAP had an initial rise in upright posture. SAP and DAP were lower after HDT
7. Liu et al. (2015a)	14 healthy men (age 30 ± 1 years; height 169 ± 1 cm; weight 62 ± 1 kg)	Longitudinal study. Subjects remained 60 days at −6° HDT. Measurements: baseline, at days 2, 17, 21, 41 and 58 of HDT, and at 6 and 12 days of recovery	SAP, DAP and MAP; HR; PP; RR intervals; SAP variability; BRS	ECG and digital infrared photoplethysmography	HR increased with HDT and stayed elevated during recovery. MAP increased late in HDT. BRS decreased late in HDT and remained reduced in recovery
8. Marshall-Goebel et al. (2018)	6 healthy men (age 41 ± 5 years)	Randomized, double-blinded crossover study. Participants remained 29 h in −12° HDT with Measurements: before and during bed rest at 4 h, 24 h, 26 h and 28.5 h of HDT	JV volume and CSA	Ultrasonography	One of the four regions evaluated of JV had a statistical significantly increased of CSA after 26 h in HDT
9. Martín-Yebra et al. (2019)	63 healthy men (mean age 30.6 years; height 178 cm; weight 73,7 kg)	Longitudinal study. There were three groups in −6° HDT: 5 days of HDT, 21 days and the group of 60 days. Measurements were made before, during HDT and after	HR; T-wave alternans: index of average alternans (IAA) and IAA normalized (IAAn)	Continuous ECG (Holter 24 h)	HR was elevated after HDT in the three groups. IAAn increased after HDT in the group of 60 days
10. Möstl et al. (2021)	24 healthy subjects (16 males and 8 females; age 33.4 ± 9.3 years; BMI 24.3 ± 2.1 kg/m²)	Before-and-after study. Participants spent 60 days in −6° HDT. Measurements: once before and on the last day of protocol	SAP and DAP; PP; HR; SV; CO; pulse arrival time; PWV; isovolumetric contraction time; aorta compliance and distensibility	Echocardiography and Doppler ultrasonography	There was an increase in the following variables: HR, DAP, arm and thigh pulse arrival time and isovolumetric contraction time. PP and SV decreased after HDT
11. Mulavara et al. (2018)	10 healthy men (age 38 ± 7 years; height 175 ± 6 cm; weight 80 ± 9 kg)	Controlled clinical trial. Subjects spent 70 days at −6° HDT bed rest. Measurements: before and four times after HDT (immediately after, 1 day, 6–7 days and 11–12 days after)	HR and MAP	ECG and digital infrared photoplethysmography	HR significantly increased after HDT.
12. Ogoh et al. (2017)	10 healthy men (age 30 ± 4 years; height 179 ± 6 cm; weight 74 ± 6 kg)	Longitudinal study. Participants remained in supine position during the protocol, which lasted 7 days: 2 days of baseline, 3 days of DI and 2 days of recovery. Measurements: once before DI, all days of DI and of recovery	SAP, DAP and MAP; HR; CO; blood flow, conductance, diameter and velocity of CA and vertebral artery	Automated sphygmomanometry, 2D-echocardiography and Doppler ultrasonography	HR increased at recovery phase. SAP was higher in DI and in recovery. CO and conductance of CA and of vertebral artery reduced during DI. CA diameters increased only in the first day of DI and CA velocity reduced during DI
13. Ogoh et al. (2020)	10 healthy men (age 27.5 ± 5.6 years; height 180.5 ± 5.2 cm; weight 77.0 ± 7.9 kg)	Longitudinal study. Volunteers spent 60 days in −6° HDT. Measurements: before HDT and on days 30 and 57	SAP, DAP and MAP; HR; blood flow and conductance of external and internal CA, of JV and of vertebral artery and vertebral vein	Digital infrared photoplethysmography, echocardiography and Doppler ultrasonography	HR increased on day 57 and external CA conductance increased on both days of HDT (30 and 57). Internal CA conductance and JV blood flow were reduced only on day 30 of HDT.
14. Orter et al. (2022)	24 healthy subjects (16 males and 8 females; age 34 ± 9 years; height 174 ± 9 cm; weight 75 ± 9 kg)	Longitudinal study. Participants past 60 days in −6° HDT. Measurements: before protocol, on days 5, 21 and 60 in bed rest and 4 days after HDT	HR; SAP and DAP; LVET; LVETi; PEP; PEPi; QS2; QS2i; PEP/LVET	Echocardiography and Doppler ultrasonography	LVETi and QS2i decreased during HDT, while PEPi and PEP/LVET had higher values compared to baseline
15. Palombo et al. (2015)	10 healthy men (age 23 ± 2 years; weight 75 ± 10 kg; BMI 23.3 ± 2 kg/m²)	Before-and-after study. Participants past 5 weeks in −6° HDT bed rest. Measurements: before protocol and within 24 h after its termination	HR; SV, SAP and DAP; aorta flow velocity integral; CA and FA IMT, diameters, end-diastolic IMT/radius, end-diastolic wall stress, beta index, diastolic and systolic peak velocity, resistive index, systolic, diastolic and mean flow per beat, systolic/diastolic flow and PWV; PP and PP index; AIx; and pressure amplification	Duplex and Doppler ultranosography, carotid applanation tonometry and PWV Complior method	SV, FA diameter and ascending aorta flow velocity integral decreased. HR increased. The ratio end-diastolic FA IMT/radius increased, and circumferential wall stress diminished. FA peak systolic and diastolic velocity increased, which diastolic velocity had a higher increase and consequent decrease in resistive index. Diastolic volumetric flow tended to decrease in CA and increase in FA. Carotid PP and PP index reduced, while pressure amplification index elevated
16. Robin et al. (2023)	18 healthy women (age 29 ± 1 years; weight 59.3 ± 1.5 kg; height 164.8 ± 1.4 cm; BMI 21.8 ± 0.4 kg/m²)	Longitudinal study. Participants spent 4 days of ambulatory baseline, followed by 5 days of DI, and 2 days of recovery. Measurements were done throughout the experiments	Calf venous compliance; HR; SAP and DAP	Occlusive air plethysmography, non-invasive blood pressure measurement and ECG	Venous compliance decreased during DI, returning to the baseline value after 1 day of recovery. HR slightly decreased, while SBP and DBP increased with DI
17. Rusanov et al. (2020)	13 healthy men (age 21–29 years)	Before-and-after study. Participants stayed 5 days in DI. Measurements: before protocol and 1 day after its termination	HR; HRV (HF, LF and VLF); stress index; RR intervals distribution: pNN50	ECG	There was a shift in the autonomic balance toward sympathetic activation. The pNN50 and total power spectrum reduced, and the stress index increased
18. Solbiati et al. (2020)	20 healthy men (mean age 36 years (28; 41–25th; 75th percentiles) and BMI 23 (23; 25)	Longitudinal study. Subjects underwent 60 days at −6° HDT. Measurements: baseline, at days 5, 21 and 58 of HDT, and at first day of recovery and after 8 days	RR and QTend oscillation amplitude, acrophase, cycle duration, minimal and maximum values and MESOR	Continuous ECG (12-lead Holter 24 h)	Maximum and minimum values of RR, and minimum values of QTend, along with both MESOR, were higher with HDT and reduced at recovery. RR and Qtend oscillation amplitude reduced during HDT and Qtend oscillation amplitude increased at recovery. QTend acrophase was higher on day 1 of recovery
19. Westby et al. (2016)	8 healthy subjects (4 males and 4 females; age 36 ± 8 years; weight 71.2 ± 2.8 kg; BMI 22.6 ± 0.9 kg/m²)	Longitudinal study. Participants underwent 11–14 ambulatory days before bed rest, 60 days of −6° HDT bed rest and 14 ambulatory days after bed rest. Measurements: before bed rest, during HDT (on days 7, 21, 31 and on day 60), and after bed rest (4 h, 3 days and 13 days)	LVSV; LVDV; LV mass; HR; SV; CO; EF; isovolumetric contraction and relaxation time; mitral E and A wave flow velocity; E/A ratio; mitral E wave annulus velocity; early and late diastolic filling velocity	2-D and 3-D echocardiography and Doppler ultrasonography	LV mass and LVDV diminished early in HDT and continued to reduce throughout the bed rest. LVSV and SV reduced on day 60 of bed rest. LV mass has a recovery after 13 days. Early and late diastolic filling velocities, together with E/A ratio and mitral E-wave flow, had an important reduction during HDT. Mitral A wave velocity and isovolumetric relaxation time increased. HR increased only after HDT

Abbreviations: AIx, augmentation index; BMI, body mass index; BPV, blood pressure variability; BRS, baroreflex sensitivity; CA, carotid artery; CO, cardiac output; CSA, cross-sectional area; DAP, diastolic arterial pressure; DI, dry immersion; ECG, electrocardiography; EF, ejection fraction; FA, femoral artery; FTA, fraction time active; HDT, head-down tilt; HF, high frequency; HR, heart rate; HRV, heart rate variability; IMT, intima-media thickness; JV, jugular vein; LF, low frequency; LV, left ventricle; LVDV, left ventricle diastolic volume; LVET, left ventricular ejection time; LVETi, left ventricular ejection time index; LVSV, left ventricle systolic volume; MAP, mean arterial pressure; MESOR, midline statistic of rhythm; mitral A, peak mitral flow during atrial systole; mitral E, peak mitral flow velocity during early filling; mitral E/A ratio, ratio between mitral E and A; PEP, pre-ejection period; PEPi, pre-ejection period index; PEP/LVET, ratio between pre-ejection period and left ventricular ejection period; pNN50, percentage of adjacent NN (R-R) intervals that differ from each other by more than 50 ms; PP, pulse pressure; PWV, pulse wave velocity; QRS-T, angle, the angle between the directions of ventricular depolarization and repolarization; QS2, total electromechanical systole; QS2i, total electromechanical systole index; RR, interval, time elapsed between two successive R-waves of the QRS, signal on the electrocardiogram; SAP, systolic arterial pressure; SV, stroke volume; SVR, systemic vascular resistance; VLF, very low frequency. (mean ± standard deviation). [minimal–maximal].

**TABLE 3:** Summary of main findings in acute simulation studies.

Acute simulation					
Authors, year published	Participants	Study design	Cardiovascular variables evaluated	Tools	Main outcomes
1. Arbeille et al. (2017)	10 healthy men (age 31.8 ± 4.1 years; height 178.8 ± 5.6 cm; weight 74.8 ± 5.6 kg; BMI 23.6 ± 1.2 kg/m²)	Before-and-after study. Subjects were exposed to 2 h of DI. Measurements were made once before and after the procedure	JV and PV volume, LVDV, LVSV, SV, CA blood flow, PV CSA, MCA and MCV flow velocity	Echography and Doppler ultrasonography	LVSV and CA blood flow reduced. JV and PV volume increased. MCV flow velocity increased but there was a large individual variability
2. Arbeille et al. (2021)	14 astronauts (11 males and 3 females; age 47 ± 6 years; BMI 26.4 ± 3 kg/m²)	Before-and-after study. Subjects were exposed to 40 min of −15° HDT. Measurements were made before the procedure in seated and supine positions and at the end of the HDT	JV volume; PV CSA; MCV velocity	Doppler ultrasonography	JV volume increased with HDT compared to baseline both in seated and supine positions
3. Ayme et al. (2014)	12 healthy men (age 34 ± 8 years; height 175 ± 6 cm; weight 70 ± 12 kg)	Crossover study. Participants went to experiments on two different days and they stayed for 1 h in head-out water immersion or 1 h in ambient air at seated position	HR; SAP, DAP, PP and MAP; SAP variability; brachial artery blood velocity and FMD; SVR; CO; SV; inferior vena cava diameter, LV and atrium diameter, and fraction shortening	ECG, digital infrared photoplethysmography and Doppler ultrasonography	CO and SV increased. SVR and SAP variability LF diminished. LV and atrial, long with inferior vena cava, increased the diameter during immersion. Brachial artery diameter and blood flow increased
4. Bimpong-Buta et al. (2020)	12 healthy subjects (7 males and 5 females; age 29 [23–31] years; height 1.77 [1.71–1.90] m; weight 80 [62–90] kg; BMI 24,5 [20–25] kg/m²)	Cross-sectional study. Volunteers participated in parabolic flights with 31 parabolas. Each parabola contained an initial hyper-G phase (1.8G), the microgravity phase and then the second hyper-G phase. Each phase lasted 22s. Measurements: Subjects were evaluated in the course of 10 consecutive parabolas, in the supine posture in five parabolas and in seated posture in the other five parabolas	SAP, DAP and MAP; HR; SV; CO; cardiac index (CO/body surface area); SVR; proportion of perfused vessels, perfused vessel density, total vessel density, number of crossing and perfused number of crossings	Digital infrared photoplethysmography and sidestream dark field microscopy	There were significant statistical changes only in seated position. SAP and DAP decreased with weightlessness. CO increased in micro and hypergravity, while HR increased only in hypergravity phase. SV diminished in microgravity
5. Carter et al. (2014)	9 healthy men (age 24.6 ± 2.0 years; height 174 ± 9 cm; weight 76 ± 9 kg; BMI 25.0 ± 1.7 kg/m²)	Cross-sectional. Participants stayed at head-out water immersion for 10 min. Measurements: baseline, during water immersion and after immersion	MAP; HR; CO; SV; MCA and PCA velocity; CA diameter and velocity	Digital infrared photoplethysmography and Doppler ultrasonography	MCA and PCA velocities increased during immersion as well as CCA diameter. MAP, CO and SV also elevated during immersion, and HR decreased
6. Chouchou et al. (2020)	10 healthy men (age 26.9 ± 5.5 years; height 181.0 ± 7.8 cm; weight 76.5 ± 8.1 kg)	Crossover study. Participants performed 3 experiments on 3 different days: −6° HDT, head-out water immersion and supine, with 30 min each. Measurements: baseline, during experiment and recovery	RR intervals; HRV (LF, HF and VLF); LF/HF ratio; SAP and DAP; BRS	ECG and digital infrared photoplethysmography	RR intervals, HF and BRS increased during all conditions, while DAP and SAP decreased. LF, VLF and total power spectrum increased at recovery. BRS returned to baseline values at recovery
7. Florian et al. (2013)	10 healthy men (age 34 ± 10 years; height 179 ± 6 cm; weight 85 ± 7 kg; BMI 26 ± 1 kg/m²)	Cross-sectional. Participants stayed at head-out water immersion for 6 h. Measurements: baseline, during water immersion and after immersion	HR; SAP, DAP and MAP; calf and forearm blood flow and vascular resistance; CO; SV; SVR; HRV LF, HF, LF/HF ratio, RMSSD and SDNN; BRS and entropy	ECG, digital infrared photoplethysmography and venous occlusion plethysmography	SAP, SV decreased, and calf blood flow decreased after DI, while venous resistance increased
8. Malhotra et al. (2021)	26 healthy subjects (20 males and 6 females; age 34.23 ± 6.98 years; height 170.22 ± 19.23 cm; weight 66 ± 8.98 kg; BMI 27.22 ± 3.76 kg/m²)	Before-and-after study. Subjects stayed 10 min at supine position and then they went to −30° HDT for 5 min. Measurements were made at the last 5 min of supine and during the HDT	RR intervals; HRV total power spectrum; RMSSD; HF, LF, VLF and LF/HF ratio	ECG	LF reduced during HDT
9. Marshall-Goebel et al. (2016)	9 healthy men (age 25 ± 2.4 years; height 183 ± 6 cm; BMI 24.1 ± 2.4 kg/m²)	Crossover study. Volunteers were exposed to 5 h of HDT in four different moments, with 5 days between the sessions. Each session started with 3 h in supine position, followed by one of the four experimental condition (−6° HDT, −12° HDT, −18° HDT and −12° HDT with 1% of CO_2_). Measurements: before each condition and at the 30 final minutes in HDT	CA, vertebral artery and JV blood flow, blood flow velocity and CSA; SAP, DAP and MAP; and HR.	MRI, digital infrared photoplethysmography and ECG.	JV CSA increased with HDT, mainly at −18°. Arteries CSA had a slight increase at −18° HDT and CA showed vasodilatation at this condition. CA blood flow velocity decreased at −6°, −12° and −18°. JV blood flow velocity also decreased at −12° and −18°. Total arterial inflow decreased from baseline to all HDT angles, with a major decrease at −12° and a trend toward increase flow from −12° to −18°. The same response was noted with total venous outflow. SAP, DAP and MAP increased from baseline at −6°; MAP and SAP differed at −12°; and MAP and DAP at −18°
10. Marshall-Goebel et al. (2019)	11 astronauts (9 males and 2 females; age 46.6 ± 6.3 years; BMI 26.4 ± 3 kg/m²)	Longitudinal study. Before spaceflight, astronauts were evaluated at seated and supine position and −15° HDT for 45 min	SV; CO; HR; SAP, DAP and MAP; JV pressure, CSA and blood flow	Blood pressure oscillometry, vein press compression sonography and Doppler ultrasonography	SV and CO increased with HDT, while HR decreased. JV CSA and pressure were significantly elevated during HDT
11. Martin et al. (2016)	11 healthy subjects (3 males and 8 females; age 39.5 [27–60] years; height 168 [157–196] cm; weight 66.6 [50.5–109.9] kg)	Cross-sectional study. Subjects participated in parabolic flights with 40 parabolas. Each parabola contained an initial hyper-G phase (1.8G), the altered gravity phase (Martian–0.29G, lunar–0.17G, or microgravity–0G) and the hyper-G phase again. Each phase lasted 20s. Measurements: before and during flight in supine position	JV pressure	Vein press compression sonography	JV pressure was elevated during 0G compared to 1G. At lunar gravity, JV pressure also increased, but less than at 0G. There was a trend, in which JV pressure increases as gravity decreases
12. Ogoh et al. (2015)	12 healthy men (age 26 ± 6 years; height 176 ± 4 cm; weight 72 ± 11 kg)	Cross-sectional study. Subjects participated in parabolic flights with 31 parabolas. The parabola contained an initial hyper-G phase (1.8G), the microgravity phase and then the second hyper-G phase. Each phase lasted 20s and there was an interval of 2 min in 1G between parabolas	SAP and DAP; HR; CO; SV; MCA velocity; total vascular conductance; cerebral perfusion pressure; central arterial pressure; thoracic impedance	Digital infrared photoplethysmography and transcranial Doppler ultrasonography	Thoracic impedance decreased during microgravity. SV, CO, central and peripheral BP and total vascular conductance increased at 0G phase. HR showed a decrease from hyper to microgravity. MCA velocity gradually decreased in microgravity phase, while cerebral pressure perfusion and carotid sinus pressure increased
13. Ogoh et al. (2018)	6 healthy men (age 24 ± 3 years; height 177 ± 2 cm; weight 69 ± 2 kg)	Cross-sectional study. Subjects participated in parabolic flights with 31 parabolas. The parabola contained an initial hyper-G phase (1.8G), the microgravity phase and then the second hyper-G phase. Each phase lasted 20s and there was an interval of 2 min in 1G between parabolas. There were two experiments: the first one a tilt table was used to determine the best angle to minimize central blood volume changes to 0G. The angles varied from 0° to −16°. The second experiment used the determined angle (−2° HDT) during the flights and the subjects remained in supine position, while the variables of interest were collected throughout the experiment	Carotid baroreflex; HR; MAP; estimated carotid sinus pressure and thoracic blood volume	Digital infrared photoplethysmography, tetrapolar high-resolution impedance monitoring and 2D-echocardiography	Experiment 1–There was a significant effect of tilt angle in thoracic blood volume and diastolic heart volume from 1G to 0G. The chosen tilt angle was −2°. Experimental 2–MAP increased during microgravity phase, as well as carotid sinus pressure and carotid baroreflex (or maximal grain)
14. Patterson et al. (2022)	20 healthy subjects (10 males: age 25 ± 5 years; BMI 23.7 ± 3.1 kg/m²; 10 females: age 24 ± 4 years; BMI 22.2 ± 2.1 kg/m²)	Crossover study. Data was collected in supine position and in HDT (−3° and −6°), and four levels of LBNP. Each condition had 5 min of duration and there were 5 min between them	JV CSA and optical attenuation	Doppler ultrasonography	Both JV CSA and optical attenuation increased with HDT compared to baseline, but there was no difference between −3° and −6° of HDT.
15. Porta et al. (2015)	13 healthy men (age 59 years)	Before-and-after study. Participants stayed for 10 min in supine position followed for 10 min in −25° HDT. Data collection was made before and at the 5 last minutes of HDT	HRV LF and HF; heart period; SAP; predictive entropy, joint transfer entropy and self-entropy	ECG	HF increased during HDT, while LF reduced. Predictive entropy and self-entropy also decreased with HDT
16. Seibert et al. (2018)	20 healthy subjects (11 males and 9 females; age 25 [22–29] years; BMI 22.7 [21.8–25.5] kg/m²)	Cross-sectional study. Subjects participated in parabolic flights with 31 parabolas. The parabola contained an initial hyper-G phase (1.8G), the microgravity phase and then the second hyper-G phase. Each phase lasted 20s and there was an interval of 2 min in 1G between parabolas and 5–8 min between each 5 ones. Measurements: data was collected during all the experiment in supine position	Central aortic and peripheral SAP and DAP; cardiac index; HR; SVR; AIx; PWV	Blood pressure oscillometry and digital infrared photoplethysmography	When shifting from 1G to 0G, central SAP and cardiac index increased, while SVR and PWV decreased. In microgravity phases, peripheral SAP decreased, and HR showed a decrease
17. Shankhwar et al. (2021)	30 healthy male (age 24 ± 3.87 years)	Crossover study. Data was collected at supine position; during 7 min of −4°, −6° and −8° HDT, with an interval of 10 min between the tilts; and at recovery	SAP, DAP and MAP; CO; SV; HRV HF, LF and LF/HF ratio	ECG and tonometry	No difference was found between baseline and −4° HDT. SAP and DAP were higher at −6° and −8°. MAP, HF, SV and CO values were elevated only at −6°. HF decreased at −8°. LF decreased only at −6°. LF/HF ratio decreased at −6° and increased at −8°. CO was higher at −8° compared to baseline and −6°
18. Watkins et al. (2017)	15 healthy subjects (8 males and 7 females)	Crossover study. Subjects were evaluated at five moments: at sitting and supine position, and at −15° HDT, with 5 min each; and 10 min of HDT with two different levels of LBNP	JV CSA; MAP; and HR	Doppler ultrasonography	JV CSA was elevated during HDT
19. Whittle et al. (2022)	12 healthy men (age 26.8 ± 2.9 years; height 179.0 ± 8.3 cm; weight 84.7 ± 18.7 kg; BMI 26.3 ± 4.9 kg/m²)	Cross-sectional study. The protocol consisted of 7 tilt angles with 12 min of duration each one: 45° HUT, 30° HUT, 15° HUT, supine (0°); −15° HDT, −30° HDT and −45° HDT. Measurements were made first in seated position and during each tilt angle	HR; SV; CO; SVR; SAP; DAP; rate pressure product; SDNN; RMSSD; HRV triangular index; LF, HF and LF/HF ratio; BRS gain	Digital infrared photoplethysmography, ECG and gas rebreathing technique	HR and SVR reduced with increasing HDT, while SV and CO elevated. Rate pressure product, SAP and DAP diminished with increasing HDT and differed from baseline. SDNN, RMSSD, HRV triangular index, LF, HF and BRS increased with HDT angles. LF/HF ratio reduced
20. Whittle et al. (2022)	12 healthy men (age 26.8 ± 2.9 years; height 179.0 ± 8.3 cm; weight 84.7 ± 18.7 kg; BMI 26.3 ± 4.9 kg/m²)	Cross-sectional study. The protocol consisted of 7 tilt angles with 12 min of duration each one: 45° HUT, 30° HUT, 15° HUT, supine (0°); −15° HDT, −30° HDT and −45° HDT. Measurements were made first in seated position and during each tilt angle	CA and JV CSA, and JV pressure	Ultrasonography and vein press compression sonography	Both CA and JV CSA, and JV pressure increased with HDT angles
21. Widjaja et al. (2015)	14 healthy men (age 28.4 ± 3.7 years; height 178.9 ± 5.5 cm; weight 77.1 ± 9.9 kg; BMI 24.0 ± 2.8 kg/m²)	Cross-sectional study. Subjects participated in parabolic flights with 31 parabolas. The parabola initiated with hyper-G phase (1.8G), the partial gravity phase (Martian–0.38G, lunar–0.16G or microgravity–0G) and then the second hyper-G phase. Each phase lasted 20s, with an interval of 2 min in 1G between parabolas and 5 min between each 6 ones. Measurements: subjects were evaluated in seated position and data was collected during normal and partial gravity phases	HRV: NN intervals, SDNN, RMSSD, pNN50, LF and HF powers and normalized units; LF/HF ratio; BPV: LF and HF powers; LF/HF ratio; SAP, DAP and MAP	ECG and digital infrared photoplethysmography	At microgravity and lunar gravity phases, NN intervals, SAP and DAP decreased. HRV and BPV were higher during 0G and lower as gravity increased, showing a negative correlation between HRV and gravity levels, for SDNN, RMSSD, pNN50, LF and HF

Abbreviations: AIx, augmentation index; BMI, body mass index; BRS, baroreflex sensitivity; CA, carotid artery; CO, cardiac output; CSA, cross-sectional area; DAP, diastolic arterial pressure; DI, dry immersion; ECG, electrocardiography; FMD, flow-mediated dilation; G, gravity; HDT, head-down tilt; HF, high frequency; HR, heart rate; HRV, heart rate variability; JV, jugular vein; LF, low frequency; LF/HF, ratio, ratio of low frequency and high frequency of heart rate variability; LV, left ventricle; LVDV, left ventricle diastolic volume; LVSV, left ventricle systolic volume; MAP, mean arterial pressure; MCA, middle cerebral artery; MCV, middle cerebral vein; MRI, magnetic resonance imaging; NN, intervals, normalized time between two successive R-waves of the QRS, signal on the electrocardiogram; PCA, posterior cerebral artery; pNN50, percentage of adjacent NN (R-R) intervals that differ from each other by more than 50 ms; PP, pulse pressure; PV, portal vein; PWV, pulse wave velocity; RMSSD, root mean square of successive differences between normal heartbeats; RR, interval, time elapsed between two successive R-waves of the QRS, signal on the electrocardiogram; SAP, systolic arterial pressure; SDNN, standard deviation of the interbeat intervals for all sinus beats; SV, stroke volume; SVR, systemic vascular resistance; VLF, very low frequency. (mean ± standard deviation). [minimal–maximal].

The outcomes were classified into three categories: anatomical, functional, and CAM outcomes, depending on the assessment method used. Anatomical outcomes were measured through imaging tests like echography, ultrasonography, and magnetic resonance imaging (MRI). Functional outcomes consisted of measures of cardiac function and blood flow, obtained through techniques like photoplethysmography, doppler ultrasonography, blood pressure oscillometry, and electrocardiogram (ECG), when used for measuring HR. When the ECG and photoplethysmography data allowed the derivation of the HRV and BRS, respectively, they were categorized as CAM.

### Meta-analysis

Meta-analysis was performed with the included articles using the Cochrane Collaboration software (Review Manager 5.4.1) (Ryan and Cochrane Consumers and Communication Review Group, 2016). In order to have a robust meta-analysis, the outcome had to be observed in at least three studies for each condition (SF, CS and AS) to be considered for the meta-analysis (Higgins et al., 2011). Outcomes that did not appear in all types of studies, were not used for meta-analysis, once the comparison is fragile. Means and standard deviations were extracted from studies. Standard deviation was computed for studies where data was presented in standard error or interquartile intervals (Ried, 2006; Higgins et al., 2011; Ryan and Cochrane Consumers and Communication Review Group, 2016).

Comparisons were conducted between the weightlessness group and the control group (not exposed to weightlessness). In studies where no control group was presented, the baseline measurements of the participants were considered as the control. For meta-analysis, the final measurement during weightlessness was used. In studies without evaluations during the intervention, values immediately after the intervention were used. Data extraction was standardized in the supine position. Data collected from the positioning of seating or functional testing were not included. The random effects model was used for analysis due to the methodological heterogeneity in the studies. Mean difference (MD) and a 95% confidence interval were calculated to measure effects.

## Results

**FIGURE 1:**
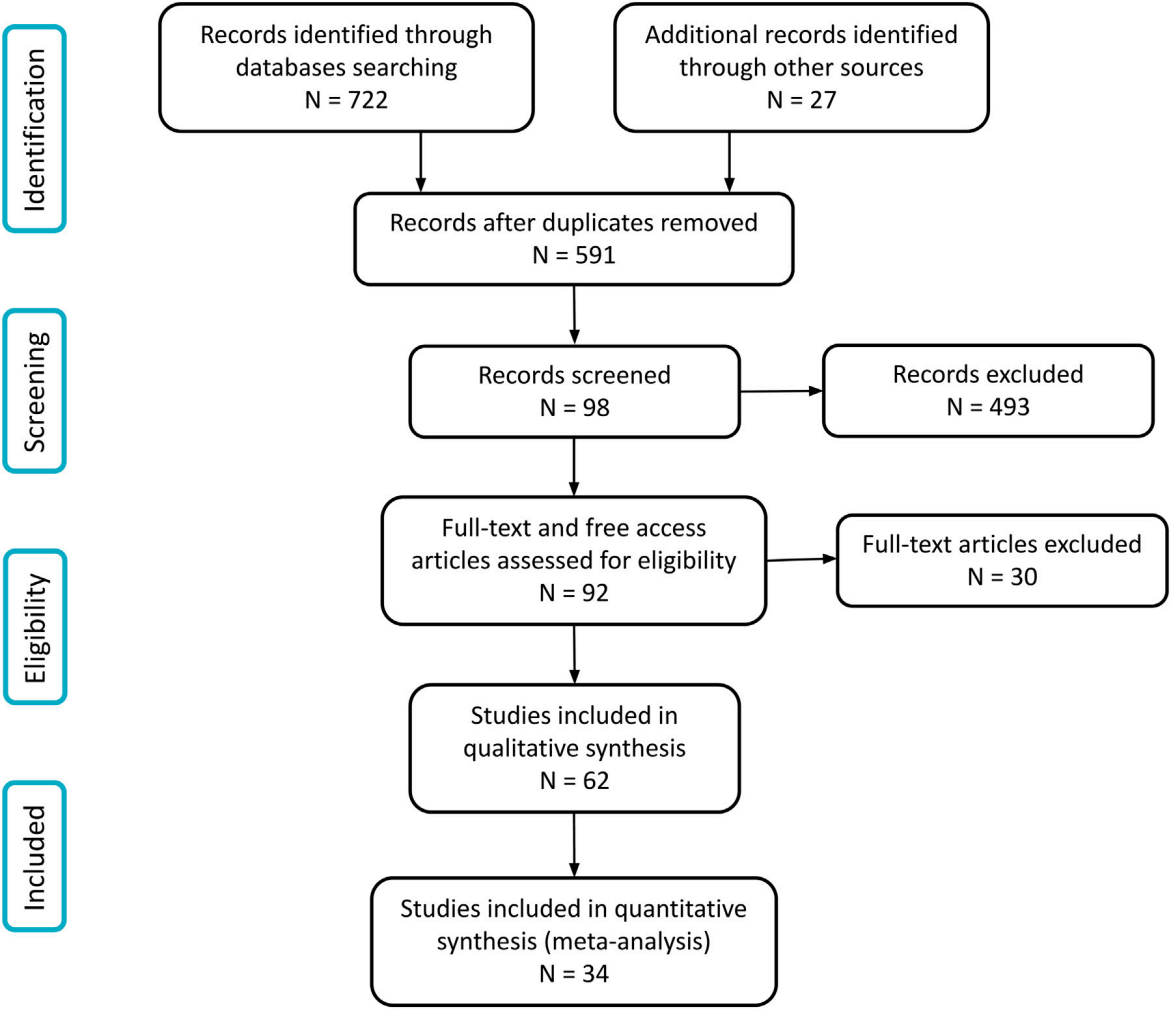
Study flow diagram.

A total of 749 studies were identified, with 722 articles retrieved from the databases, and a further 27 articles from other sources, mainly through a review of the references used in the included articles from the databases. After the database search update, two more studies were included. Sixty-two articles were included in this review (Figure 1), in which 58 used exclusively one type of weightlessness model, and other 4 studies utilized both SF and CS or SF and AS. A summary table was created as a qualitative synthesis for each type of study, presenting the main findings of all the 62 included studies. For the quantitative synthesis, the meta-analysis, only 34 of these studies were included.

Twenty-six studies were included in the spaceflight condition, with an average duration of 163.7 days (about 5 and a half months) and ranging from 12 to 327 days (about 10 and a half months). Nineteen studies were considered as CS. Among these, 16 studies used HDT, which 15 considered −6° of inclination, and one study was conducted with −12° HDT. Only three studies used DI. The average duration of CS studies was 37.8 days (ranging between one to 70 days). For AS, 21 studies were included. Of these studies, six employed parabolic flight, with variations in the number of parabolas used; each parabola featured a mean microgravity phase lasting 20 s. In eleven studies, HDT was utilized, with a range of 5 min–6 h of exposure. DI was utilized only in one study, while an alternative DI approach, referred to as “head-out water immersion” (WI), was used in three other studies, with exposure periods ranging from 10 min to 6 h.

All the included articles and their main findings of studies are summarized and presented on Tables 1–3. It was considered only the weightlessness group without any countermeasure or other intervention.

### Population

In this review, a total of 963 participants were enrolled across all three conditions of weightlessness investigated. This value was calculated considering the sample size (n) of each study; therefore, it is possible that there are overlapping participants among the studies. Of the participants, 346 were involved in the SF studies. The sample size ranged from one to 85 subjects, with a mean of 13 (10 male and 3 female) astronauts per study. The CS studies involved 329 participants, with a varying sample size ranging from 6 to 63 individuals and a mean of 17 (15 male and 2 female) subjects per study. Two hundred and eighty-eight participants took part in the AS studies, with the sample size varying from 6 to 30 per study, and a mean of 14 participants (11 males and 3 females) per study. Table 4 displays the demographic characteristics of the participants.

**TABLE 4:** Participants’ demographic characterization.

	Subjects per study	Sex (female/male ratio)	Age (years)	Height (m)	Weight (Kg)	BMI (Kg/m^2^)
SF	13.3 ± 15.6	0.29 ± 0.23	46.6 ± 3.0	1.75 ± 0.03	76.8 ± 6.0	25.1 ± 1.4
CS	17.3 ± 13.0	0.14 ± 0.29	31.5 ± 5.0	1.75 ± 0.05	71.6 ± 6.0	23.5 ± 1.3
AS	13.7 ± 5.8	0.33 ± 0.63	31.9 ± 9.5	1.77 ± 0.04	75.9 ± 6.6	24.2 ± 1.9
Total	14.6 ± 12.4	0.26 ± 0.42	37.2 ± 9.6	1.76 ± 0.04	74.8 ± 6.5	24.3 ± 1.7

Demographic characteristics for each study type and considering the three types together. Data are presented in mean ± standard deviation. Abbreviations: AS, acute simulation; BMI, body mass index; CS, chronic simulation; SF, spaceflight.

### Cardiac outcomes

#### Cardiac function

*Heart rate (HR):* HR had a significant increase during spaceflight (Xu et al., 2013; Liu Z. et al., 2015; Lee et al., 2015; Hughson et al., 2016; Mulavara et al., 2018; Fu et al., 2019; Hoffmann et al., 2019; Wood et al., 2019; Lee et al., 2020; Iwasaki et al., 2021; Otsuka et al., 2022), and right after CS (Adami et al., 2013; Liu J. et al., 2015; Palombo et al., 2015; Westby et al., 2016a; Ogoh et al., 2017; Mulavara et al., 2018; Martín-Yebra et al., 2019; Ogoh et al., 2020; Möstl et al., 2021). Conversely, some studies with AS showed a decrease in HR (Carter et al., 2014; Ogoh et al., 2015; Seibert et al., 2018; Marshall-Goebel et al., 2019; Whittle et al., 2022).

*Blood pressure (BP):* during SF, two studies showed reduction of systolic arterial pressure (SAP) (Norsk et al., 2015; Fu et al., 2019), diastolic arterial pressure (DAP) (Norsk et al., 2015; Arbeille et al., 2017a) and mean arterial pressure (MAP) (Norsk et al., 2015; Fu et al., 2019), one reported an increase in BP in general (Lee et al., 2015) and also only study reported an increase in DAP (Wood et al., 2019), and the others remained unchanged (Hughson et al., 2016; Mulavara et al., 2018; Hoffmann et al., 2019; Marshall-Goebel et al., 2019; Lee et al., 2020; Iwasaki et al., 2021). Regarding CS models, SAP decreased in two studies (Arzeno et al., 2013; Linnarsson et al., 2015) and increased in other two (Ogoh et al., 2017; Robin et al., 2023). DAP was diminished in one study (Arzeno et al., 2013) and elevated in other two as well (Möstl et al., 2021; Robin et al., 2023). MAP also decreased in one study (Adami et al., 2013) and increased in another one (Liu J. et al., 2015). In AS, SAP, DAP and MAP were reduced in six studies (Florian et al., 2013; Widjaja et al., 2015; Seibert et al., 2018; Bimpong-Buta et al., 2020; Chouchou et al., 2020; Whittle et al., 2022), while five studies reported a significant increase (Carter et al., 2014; Ogoh et al., 2015; Marshall-Goebel et al., 2016; Ogoh et al., 2018; Shankhwar et al., 2021). Pulse pressure reduced in one study with SF (Fu et al., 2019) and in two studies with CS (Palombo et al., 2015; Möstl et al., 2021). In addition, two studies found higher isovolumetric contraction (Möstl et al., 2021) and relaxation time (Westby et al., 2016a), suggesting central systolic and diastolic dysfunction.

*Stroke volume (SV):* Five studies evaluated SV in SF (Lee et al., 2015; Norsk et al., 2015; Hughson et al., 2016; Marshall-Goebel et al., 2019; Lee et al., 2020). Two studies reported an increase in SV (Norsk et al., 2015; Marshall-Goebel et al., 2019), while the other two studies reported a reduction in SV (Lee et al., 2015; Lee et al., 2020). In CS studies, SV was diminished in all studies with this outcome (Adami et al., 2013; Palombo et al., 2015; Westby et al., 2016a; Möstl et al., 2021), while in AS, six studies reported an increase (Ayme et al., 2014; Carter et al., 2014; Ogoh et al., 2015; Marshall-Goebel et al., 2019; Shankhwar et al., 2021; Whittle et al., 2022), and two presented lower SV values compared to baseline (Florian et al., 2013; Bimpong-Buta et al., 2020).

*Cardiac output (CO):* CO increased in three of the five SF studies (Norsk et al., 2015; Marshall-Goebel et al., 2019; Lee et al., 2020) and decreased in only one study (Lee et al., 2015). CO was markedly decreased in two studies using CS (Adami et al., 2013; Ogoh et al., 2017) and was elevated in seven studies with AS (Ayme et al., 2014; Carter et al., 2014; Ogoh et al., 2015; Marshall-Goebel et al., 2019; Bimpong-Buta et al., 2020; Shankhwar et al., 2021; Whittle et al., 2022).

#### Cardiac dimension and morphology

One study with AS identified increments of the left ventricle end-diastolic diameter and atrium diameter (Ayme et al., 2014). Studies with CS described a decrease of left ventricle mass and blood volume, both in systole and diastole (Westby et al., 2016a; Orter et al., 2022).

#### Cardiac electrophysiology

Two CS studies identified alterations in the cardiac electrophysiology, including a reduction in T-wave area, an increase in apex and amplitude, and a decrease in QRS-T angle (Caiani et al., 2013; Martín-Yebra et al., 2019). Another study, conducted with SF, investigated atrial alterations and found increased left atrial blood volume post-flight (Khine et al., 2018). Additionally, some derivations of the electrocardiogram (ECG) showed a decrease in P-wave amplitude (Khine et al., 2018). One astronaut had a significant increase in supraventricular ectopic beats but no evidence of atrial fibrillation (Khine et al., 2018).

### Vascular outcomes

#### Vascular hemodynamics and morphology

Middle cerebral artery (MCA) velocity increased in one study with SF (Iwasaki et al., 2021) and in one with AS (Carter et al., 2014). In addition, in AS, one study had a decrease in MCA velocity (Ogoh et al., 2015). Both carotid artery and femoral artery cross-sectional area (CSA) and intima-media thickness (IMT) increased in space (Arbeille et al., 2016; Arbeille et al., 2017a). In AS, two studies identified an increase in carotid artery CSA as well (Whittle and Diaz-Artiles, 1985; Marshall-Goebel et al., 2016), while one CS study found an increase in carotid artery diameter (Ogoh et al., 2017). Jugular vein CSA and volume were higher in all the three conditions (Whittle and Diaz-Artiles, 1985; Arbeille et al., 2015; Marshall-Goebel et al., 2016; Arbeille et al., 2017b; Watkins et al., 2017; Marshall-Goebel et al., 2018; Marshall-Goebel et al., 2019; Arbeille et al., 2021; Patterson et al., 2022; Pavela et al., 2022). Systemic vascular resistance increased in one study in space (Lee et al., 2020) and decreased in other studies with SF, CS and AS (Adami et al., 2013; Ayme et al., 2014; Norsk et al., 2015; Seibert et al., 2018; Whittle et al., 2022). Additionally, aortic pulse wave velocity decreased in AS (Seibert et al., 2018), while no differences were observed in SF and CS (Palombo et al., 2015; Hoffmann et al., 2019; Möstl et al., 2021).

### Cardiovascular autonomic modulation (CAM) outcomes

#### CAM in frequency domain

The cardiovascular autonomic modulation was influenced by microgravity. During SF, the low frequency (LF) of the heart rate variability (HRV) reduced in two of six studies (Xu et al., 2013; Yamamoto et al., 2015), and increased in one (Otsuka et al., 2022), while in the other three studies there was no difference (Vandeput et al., 2013; Otsuka et al., 2015; Otsuka et al., 2016). For high frequency (HF) of HRV, four studies showed a reduction (Vandeput et al., 2013; Xu et al., 2013; Yamamoto et al., 2015; Otsuka et al., 2022), and two had no difference (Otsuka et al., 2015; Otsuka et al., 2016). The LF/HF ratio was reduced in one study (Vandeput et al., 2013). In CS, two studies showed a reduction of the HF (Arzeno et al., 2013; Liang et al., 2014) and one of the LF (Liang et al., 2014), and LF increased in one study (Arzeno et al., 2013). In AS, five studies showed an increase in the HF band of HRV (Porta et al., 2015; Widjaja et al., 2015; Chouchou et al., 2020; Shankhwar et al., 2021; Whittle et al., 2022). LF had an increase in three studies (Widjaja et al., 2015; Chouchou et al., 2020; Whittle et al., 2022) and a decrease in other three studies (Porta et al., 2015; Malhotra et al., 2021; Shankhwar et al., 2021). The LF/HF ratio was diminished in two studies (Shankhwar et al., 2021; Whittle et al., 2022).

#### CAM in time domain

HRV in time domain in space had decrease in the following variables: pNN50 (percentage of adjacent NN (R-R) intervals that differ from each other by more than 50 ms) (Vandeput et al., 2013; Otsuka et al., 2022), SDRR (standard deviation of the R-R intervals) (Xu et al., 2013; Yamamoto et al., 2015), SDARR (standard deviation of the set of averaged RR intervals) (Xu et al., 2013; Yamamoto et al., 2015), and RMSSD (root mean square of successive differences between normal heartbeats) (Vandeput et al., 2013; Xu et al., 2013; Yamamoto et al., 2015; Otsuka et al., 2022). For CS studies, pNN50 was reduced (Rusanov et al., 2020), and during AS, pNN50, SDNN and RMSSD were increased (Widjaja et al., 2015; Whittle et al., 2022).

#### Baroreflex sensitivity

Blood pressure variability did not change during SF and CS (Arzeno et al., 2013; Fu et al., 2019) but increased during AS (Widjaja et al., 2015). Baroreflex sensitivity decreased in CS (Arzeno et al., 2013; Liu J. et al., 2015) but increased in AS in two studies (Chouchou et al., 2020; Whittle et al., 2022). Most studies showed no changes in RR intervals (Caiani et al., 2013; Vandeput et al., 2013; Liu J. et al., 2015; Rusanov et al., 2020; Malhotra et al., 2021), but one CS study identified a reduction (Arzeno et al., 2013) and another AS study showed an increase (Chouchou et al., 2020).

### Meta-analysis

Meta-analysis was conducted for the following variables: HR, SAP, CO and SV. The results of SF and CS are presented both separately and grouped due to their chronic exposure characteristics. Nevertheless, AS data was presented separately.

**FIGURE 2:**
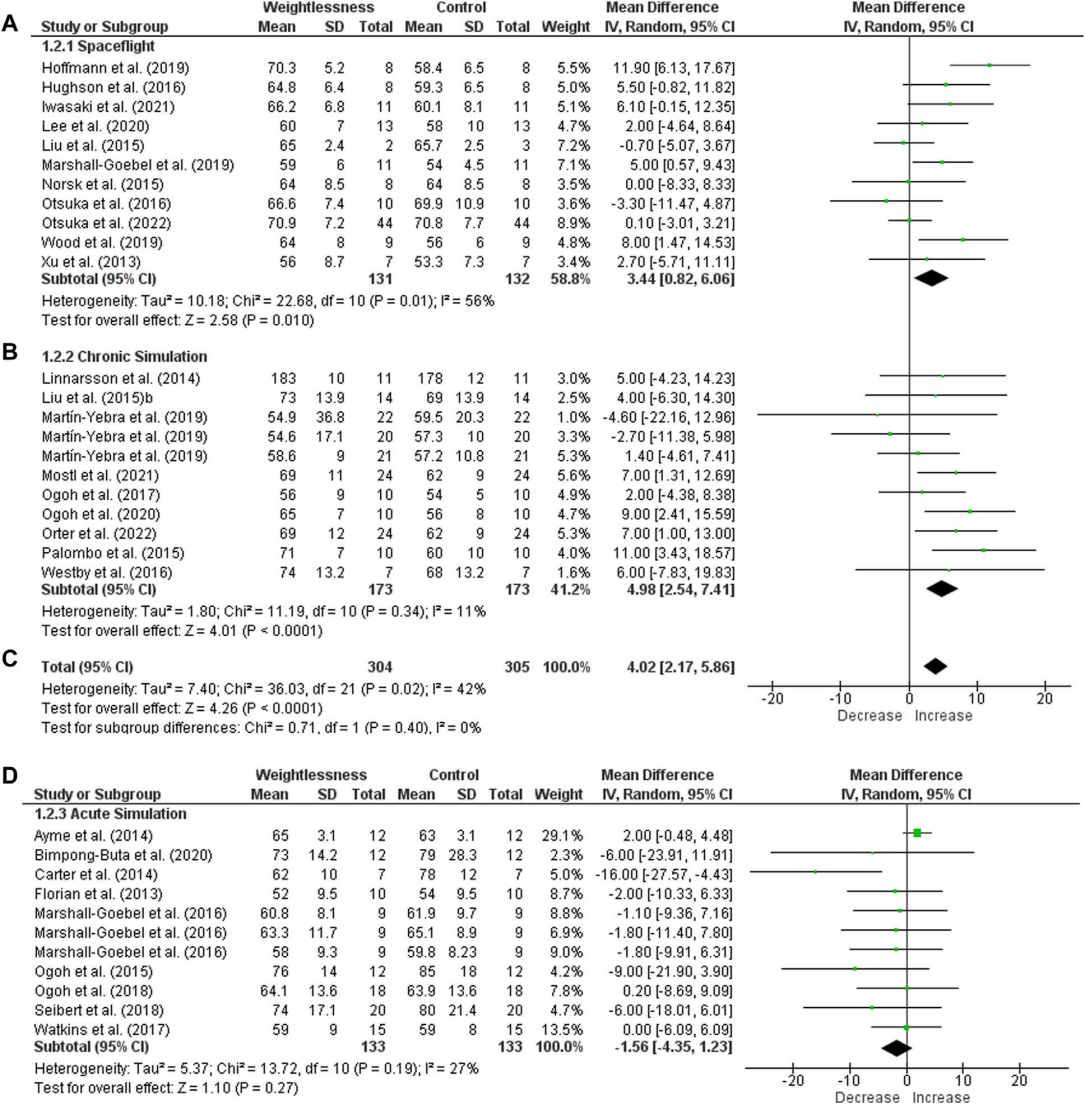
Forest plot of Heart Rate. Effect sizes after weightlessness exposure compared to control group or baseline. **(A)** Spaceflight; **(B)** Chronic Simulation; **(C)** Total effect size of the Spaceflight and Chronic Simulation; **(D)** Acute Simulation. The left side of the forest plot represents a decrease in the value of the outcome. The right side of the forest plot represents an increase in the value of the outcome.

Eleven studies with HR in SF were included in meta-analysis (Figure 2). Heterogeneity was I^2^ = 56%, and it presented a significant effect size (131 subjects, MD = 3.44, 95% CI 0.82 to 6.06, Z = 2.58, *p* = .010). For CS, nine studies with HR were included. One of them was repeated three times in the graphic, once it had three groups with different duration of weightlessness simulation. Heterogeneity was I^2^ = 11%, and it showed a significant effect size (173 subjects, MD = 4.98, 95% CI 2.54 to 7.41, Z = 4.01, *p* < .0001). When HR was grouped for SF and CS meta-analysis showed a significant increase (effect size: MD = 4.02, 95% CI 2.17 to 5.86, Z = 4.26, *p* < .0001), with low heterogeneity (I^2^ = 42%). Concerning the HR in AS studies, nine studies were included, and one was repeated three times as well as in the CS group. Heterogeneity was low (I^2^ = 27%) with no effect size (133 subjects, MD = −1.56, 95% CI −4.35 to 1.23, Z = 1.10, *p* = .27).

**FIGURE 3:**
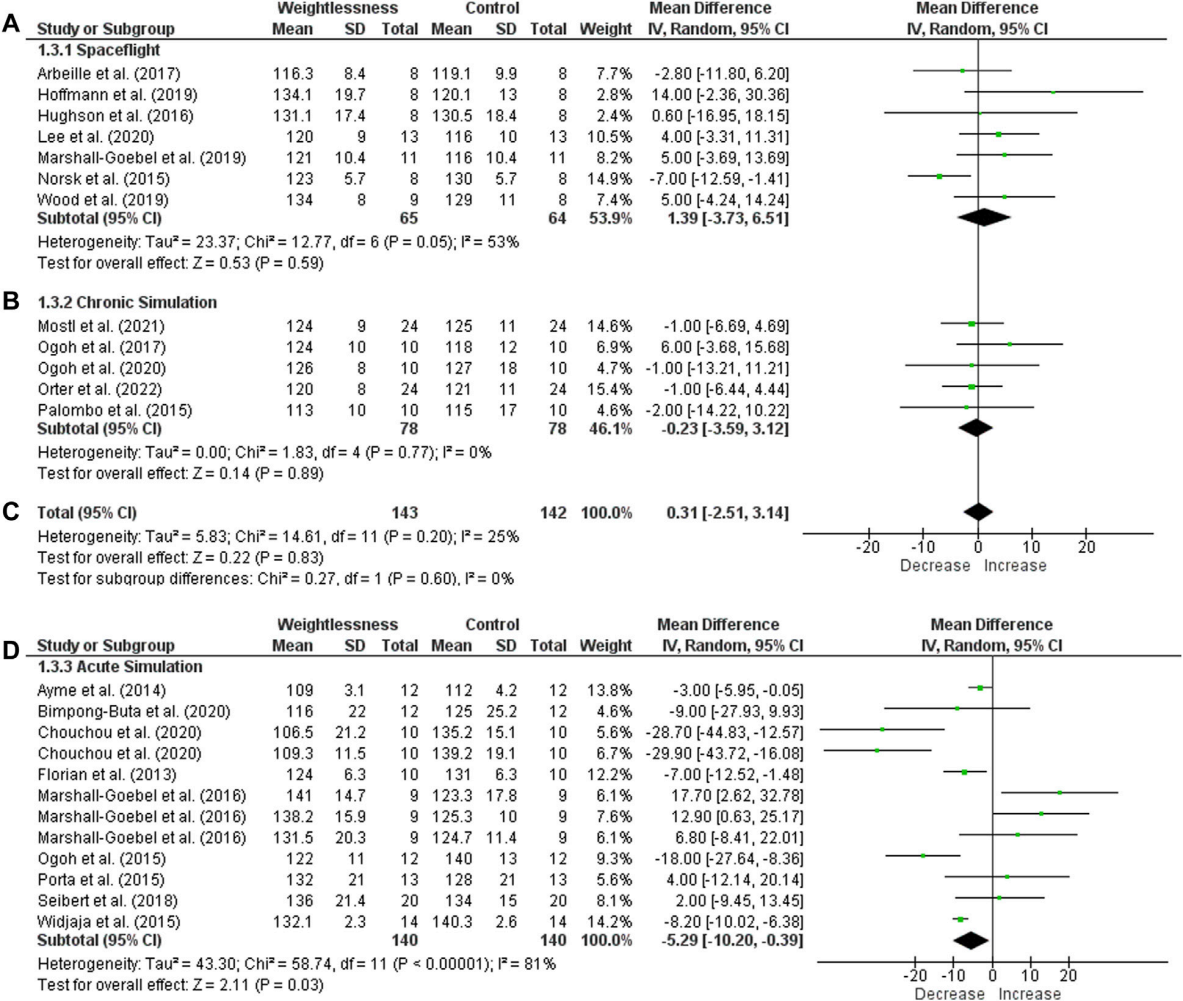
Forest plot of Systolic Arterial Pressure. Effect sizes after weightlessness exposure compared to control group or baseline. **(A)** Spaceflight; **(B)** Chronic Simulation; **(C)** Total effect size of the Spaceflight and Chronic Simulation; **(D)** Acute Simulation. The left side of the forest plot represents a decrease in the value of the outcome. The right side of the forest plot represents an increase in the value of the outcome.

Considering SF, seven studies investigated SAP and were included for meta-analysis (Figure 3). Heterogeneity was 53%, and it had no significant effect size (65 subjects, MD = 1.39, 95% CI −3.73 to 6.51, Z = 0.53, *p* = .59). About CS studies, five were included, and SAP also had no significant difference (78 subjects, MD = −0.23, 95% CI −3.59 to 3.12, Z = 0.14, *p* = .89), with heterogeneity of I^2^ = 0%. When both SF and CS were grouped, heterogeneity was I^2^ = 25% and had no significant effect size (143 subjects, MD = 0.31, 95% CI −2.51 to 3.14, Z = 0.22, *p* = .83). Nine of AS studies were considered, and they showed a significant decrease (effect size: MD = −5.29, 95% CI −10.20 to −0.39, Z = 2.11, *p* = .03), but with high heterogeneity (I^2^ = 81%).

**FIGURE 4:**
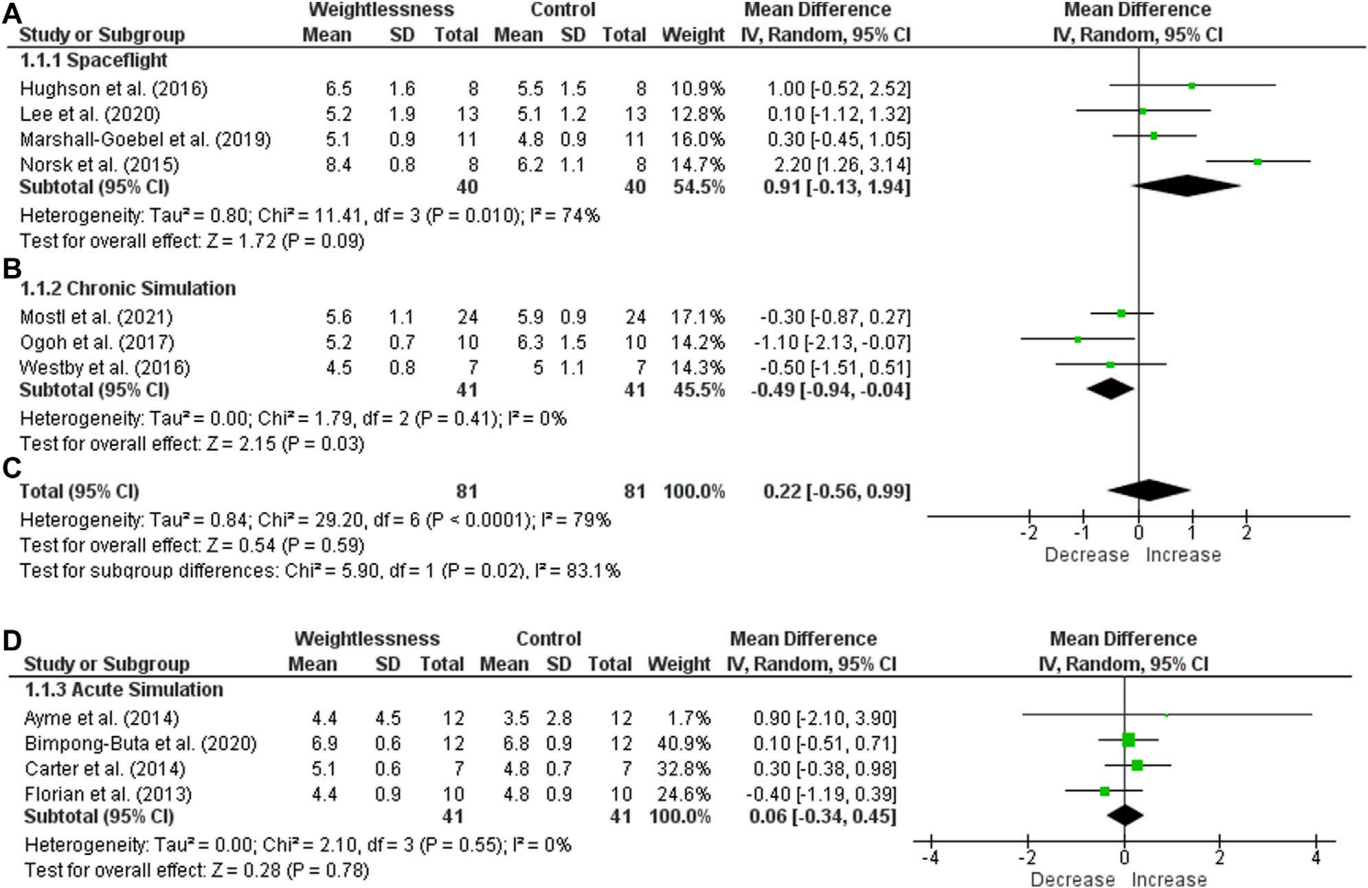
Forest plot of Cardiac Output. Effect sizes after weightlessness exposure compared to control group or baseline. **(A)** Spaceflight; **(B)** Chronic Simulation; **(C)** Total effect size of the Spaceflight and Chronic Simulation; **(D)** Acute Simulation. The left side of the forest plot represents a decrease in the value of the outcome. The right side of the forest plot represents an increase in the value of the outcome.

Regarding CO in SF, four studies were included and there was no statistical significance (heterogeneity of I^2^ = 74%, and effect size of 40 subjects, MD = 0.91, 95% CI −0.13 to 1.94, Z = 1.72, *p* = .09). There was a significant decrease of CO in CS, with 3 studies included (I^2^ = 0% and effect size: MD = −0.49, 95% CI -0.94 to −0.04, Z = 2.15, *p* = .03). The total heterogeneity of the groups SF and CS was high (I^2^ = 79%) with no significant effect size (81 subjects, MD = 0.22, 95% CI −0.56 to 0.99, Z = 0.54, *p* = .59), as seen in Figure 4. No difference was found for AS studies (heterogeneity of I^2^ = 0% and no significant effect size of 41 subjects, MD = 0.06, 95% CI −0.34 to 0.45, Z = 0.28, *p* = .78), with four studies included.

**FIGURE 5:**
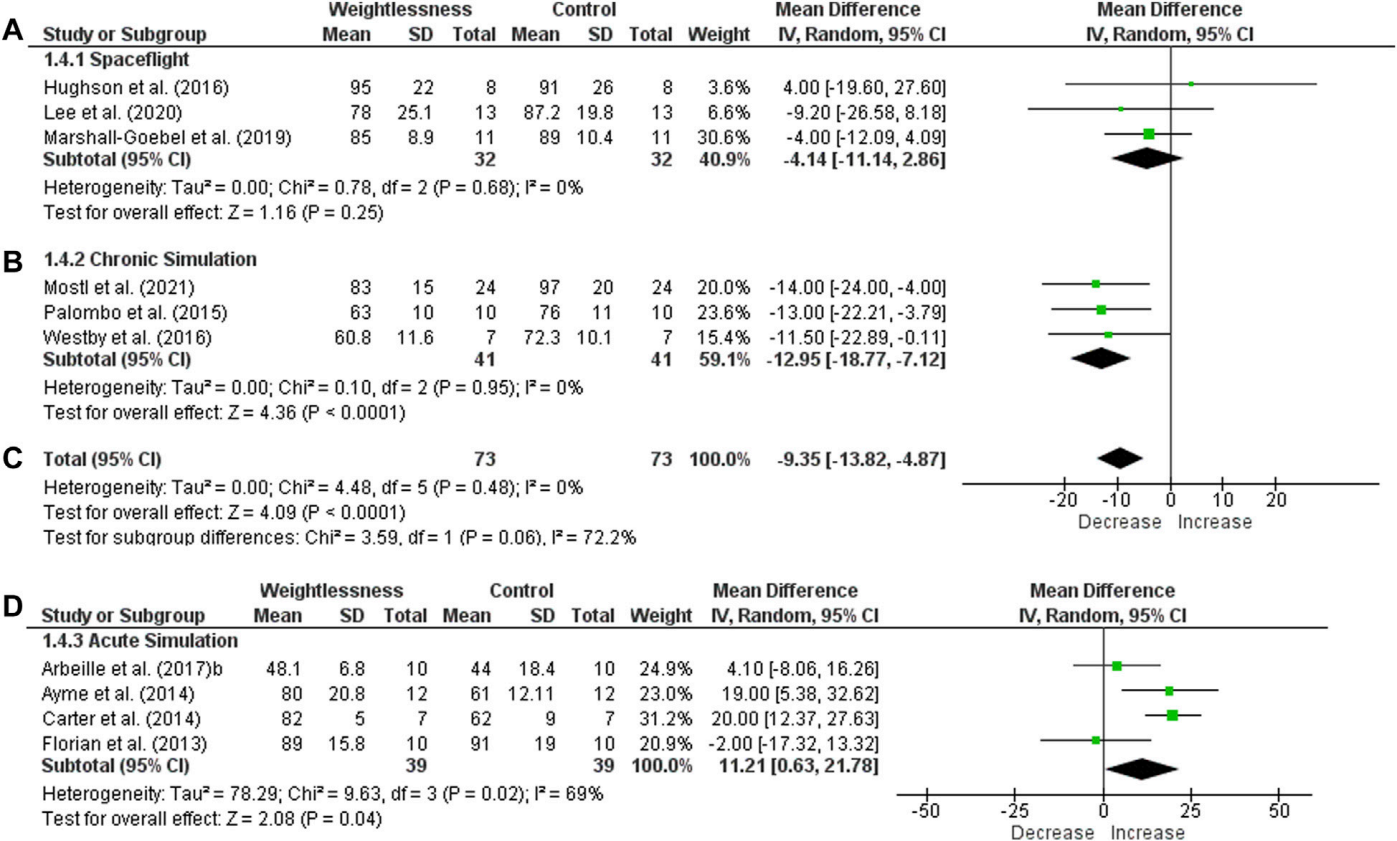
Forest plot of Stroke Volume. Effect sizes after weightlessness exposure compared to control group or baseline. **(A)** Spaceflight; **(B)** Chronic Simulation; **(C)** Total effect size of the Spaceflight and Chronic Simulation; **(D)** Acute Simulation. The left side of the forest plot represents a decrease in the value of the outcome. The right side of the forest plot represents an increase in the value of the outcome.

Finally, SV was unchanged in space (heterogeneity of I^2^ = 0% and effect size of 32 subjects, MD = −4.14, 95% CI −11.14 to 2.86, Z = 1.16, *p* = .25) (Figure 5). SV was significantly reduced in CS (heterogeneity of I^2^ = 0% and significant effect size of 41 subjects, MD = −12.95, 95% CI −18.77 to −7.12, Z = 4.36, *p* < .0001). For the two groups (SF and CS), heterogeneity was I^2^ = 0% and there was significant effect size, indicating a decrease of SV (73 subjects, MD = −9.35, 95% CI −13.82 to −4.87, Z = 4.09, *p* < .0001). About AS studies, four of ten studies were included in meta-analysis, and SV had a significant increase (heterogeneity of I^2^ = 69% and effect size of 39 subjects, MD = 11.21, 95% CI 0.63 to 21.78, Z = 2.08, *p* = .04).

## Discussion

This study evaluated the impact of microgravity on the cardiovascular system of healthy subjects, specifically focusing on structural, physiological, and functional properties. The study conducted a systematic review of existing literature and a meta-analysis of both acute and chronic effects of weightlessness on the cardiovascular system, providing new evidence to support medical research in space. It was observed that weightlessness has an impact on the cardiovascular system, causing changes to cardiac function, vascular health, and cardiovascular autonomic modulation.

### Advantages and disadvantages of each model of microgravity

Considering the impact of weightlessness on the cardiovascular system, there were some specificities inherent for each study condition, with corresponding strengths and limitations for data interpretation. The main advantage of SF research lies in real environment, which promotes trustworthy findings about microgravity effects. However, several aspects must be considered before data generalization. For instance, the small sample size of Space Shuttle and Space Station missions due to their expensive nature and the need for a specific and well-trained crew, mostly consisting of male subjects, means that the results are highly influenced by inter-individual variability. Additionally, astronauts are subject to various factors associated with spaceflight, including exposure to cosmic radiation, confinement, sleep deprivation, altered nutrition, and high concentrations of CO_2_ on the spacecraft. Equally significant, astronauts often partake in multiple studies, potentially impacting the outcomes under investigation (Lee et al., 2015; Hughson et al., 2016; Fu et al., 2019).

Interestingly, chronic models that simulate spaceflight (DI and HDT) demonstrate similar outcomes to those seen in long-duration spaceflights, as observed in the meta-analysis. These models offer certain advantages, such as a controlled environment that allows for a concentrated focus on the primary outcomes with minimal interference from external factors, thereby enabling testing of countermeasures to ensure their effectiveness in space missions (Hargens and Vico, 1985). It is impossible to fully eliminate the effects of gravitational force, making the results of experiments conducted in space less reliable and reproducible. Additionally, chronic simulations share certain drawbacks with spaceflight studies, including a small sample size and a predominance of male volunteers. The literature reports high costs associated with long protocols, including those lasting 70 days in HDT (Shen and Frishman, 2019). This kind of study typically requires numerous specialized professionals to guarantee safety measures, along with multiple concurrent research experiments that involve enrolling the same individuals (Shen and Frishman, 2019).

Acute models of microgravity simulation allow for more intense and focused observation of effects while avoiding the accommodation effect. Though exposure time is brief in comparison to actual spaceflight, acute effects are valuable for representing the initial phase of flight (Shen and Frishman, 2019). DI and HDT acute simulations offer practical and low-cost advantages. It is important to note that the Earth’s gravitational influence, akin to CS, is present in both types of models. It has the potential to affect the outcomes (Ploutz-Snyder, 2016). Parabolic flight enables a real microgravity experience on Earth. However, due to the brief duration of the parabolic phase (typically lasting only 20 s), conducting cardiovascular assessments can be challenging. Other factors that may impact microgravity responses include the hypergravity phase, small sample sizes, and the higher likelihood of motion sickness among participants, for which medication may be necessary (Shen and Frishman, 2019).

### Cardiovascular impairments and astronaut physiology

**FIGURE 6:**
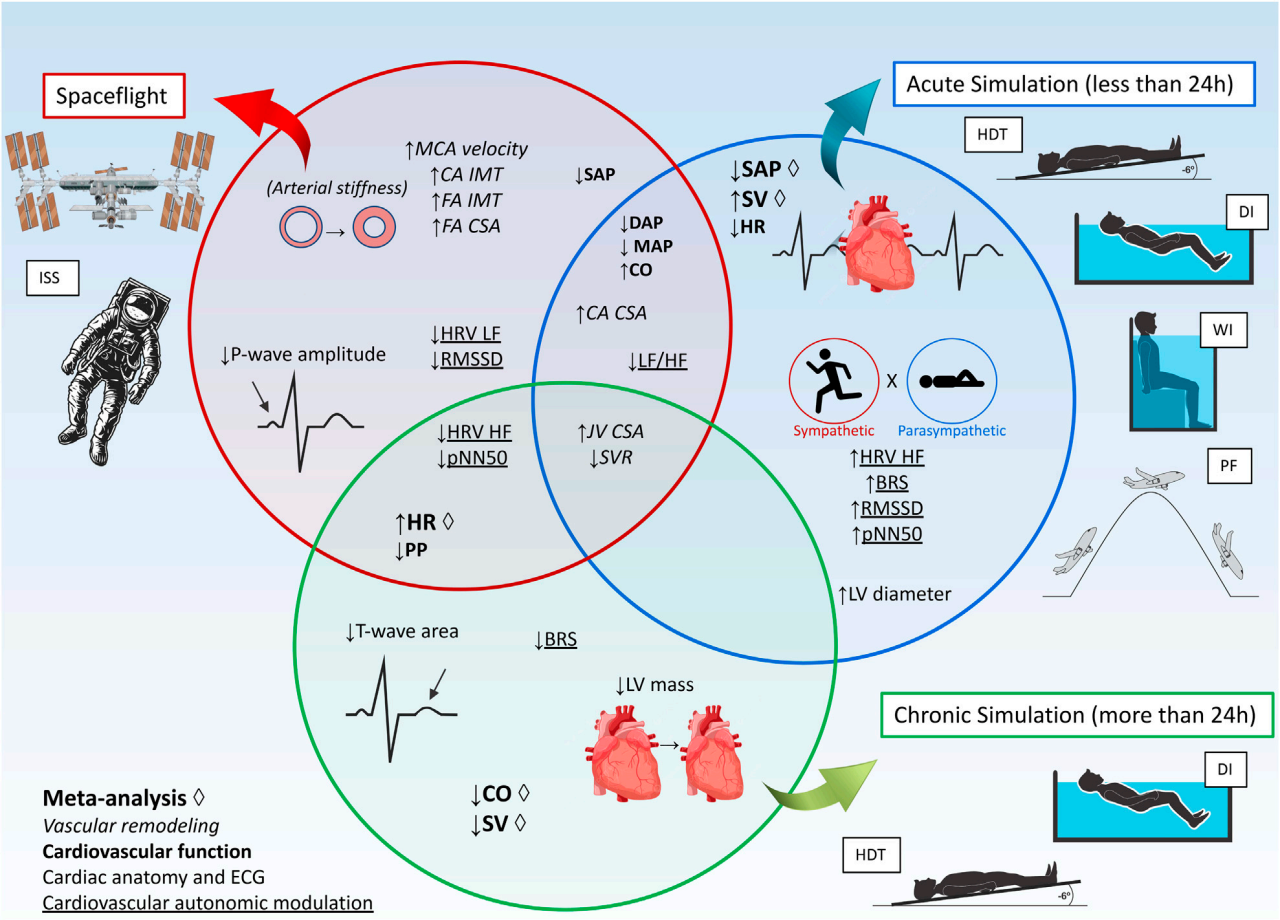
Summary of results. Abbreviations: BRS, baroreflex sensitivity; CA, carotid artery; CO, cardiac output; CSA, cross-sectional area; DAP, diastolic arterial pressure; DI, dry immersion; ECG, electrocardiography; FA, Femoral artery; HDT, head-down tilt; HF, high frequency; HR, heart rate; HRV, heart rate variability; IMT, Intima-media thickness; ISS, International Space Station; JV, jugular vein; LF, low frequency; LF/HF ratio, ratio of low frequency and high frequency of heart rate variability; LV, left ventricle; MAP, mean arterial pressure; MCA, middle cerebral artery; PF, Parabolic Flight; pNN50, percentage of adjacent NN (R-R) intervals that differ from each other by more than 50 ms; PP, pulse pressure; RMSSD, root mean square of successive differences between normal heartbeats; SAP, systolic arterial pressure; SV, stroke volume; SVR, systemic vascular resistance; WI, Head-out water immersion.

During SF, astronauts suffer from generalized deconditioning, which impacts different systems such as musculoskeletal, immune, cardiovascular, vestibular systems, among others. Specifically, weightlessness can modify different aspects of the cardiovascular system, including fluid balance, renal hemodynamics, vascular adaptation, and cardiovascular autonomic neural control (Aubert et al., 2005). This review has identified cardiac remodeling, as an indicative of atrophy, decreased SV, and impaired baroreflex sensitivity as significant findings. Previous studies during Neurolab and Spacelab Missions had similar findings (Cooke et al., 1985; Perhonen et al., 1985; Levine et al., 2002; Eckberg et al., 2010). A summary of the main results is presented in Figure 6.

During the first 24 h in space, the absence of gravity results in a decrease in intrathoracic pressure due to chest expansion. Studies of European Space Agency parabolic flight campaigns (Pump et al., 1985; Videbaek and Norsk, 1985) have suggested an increase in transmural cardiac pressure, cardiac preload, and atrial stretching, that could be primarily caused by an elevation in plasma volume in the trunk (Aubert et al., 2005; Norsk, 2020). Atrial stretching results in an elevation of atrial natriuretic peptide levels. This, in conjunction with decreased blood vessel tension due to the absence of gravity, leads to vasodilation and enhanced permeability (Norsk, 2020). Moreover, based on the Frank Starling mechanism, distension of the cardiac chambers enhances the final diastolic volume, thereby increasing the SV. The present review’s meta-analysis observed a considerable rise in SV in AS, consistent with existing literature (Aubert et al., 2005; Norsk, 2020).

Increased SV is typically followed by an expected increase in CO shortly after exposure to microgravity. However, the meta-analysis displayed no changes to CO or HR, and a decrease in SAP during AS. Lower SAP values can trigger greater activation of the Renin Angiotensin Aldosterone System, which results in the reabsorption of sodium and water, and an increase in vascular resistance in arterioles as an attempt to regulate SAP (Aubert et al., 2005). Furthermore, the initial increase in plasma volume in the trunk leads to an attempt to return to baseline state through a greater process of natriuresis and diuresis (Norsk, 2020). Chronically, this process leads to dehydration (Norsk, 2020).

Extended exposure to weightlessness significantly decreases plasma volume, potentially due to systemic vascular resistance and peripheral arterial vasoconstriction (Norsk, 2020). This may prompt high venous compliance, which contributes to the maintenance of SAP reduction during prolonged weightlessness as reflected in the majority of the studies included in this review (Norsk, 2020). The reduction of blood pressure, in general, can be observed in both SF and AS, as illustrated in Figure 6. According to our meta-analysis findings, SAP tends to stabilize over time, while SV decreases after CS (the same pattern observed during SF). Furthermore, our results show a decrease in left ventricular mass and blood volume, along with systemic hypovolemia and shrinkage of the cardiac cavity. These changes may contribute to systolic and diastolic dysfunction (Caiani et al., 2013; Westby et al., 2016a; Martín-Yebra et al., 2019). Literature suggests that cardiac atrophy and deconditioning occur in both SF and CS conditions as a form of adaptation (Dorfman et al., 1985a; Dorfman et al., 1985b; Perhonen et al., 1985; Westby et al., 2016), similar to the effects of aging, sedentary behavior, and immobility (Hart, 2023; Malhan et al., 2023). Of particular concern for cardiac deconditioning is the slow process of reconditioning after return to Earth (Solbiati et al., 2020).

Electrocardiographic alterations, such as reduced P-wave amplitude and T-wave area, should not be overlooked since they can pose a potential risk to atrial fibrillation and arrhythmia (Caiani et al., 2013; Khine et al., 2018). Also, these alterations can be associated with fluid loss and hypovolemia, which may also affect cardiovascular autonomic modulation (Caiani et al., 2013; Martín-Yebra et al., 2019). Our findings are consistent with the previous literature and other studies not included in this review, which indicates a decrease in both sympathetic and parasympathetic components of HRV, however, there is a significant reduction in parasympathetic responses, resulting in a relative predominance of sympathetic activity (Cooke et al., 1985; Iwasaki et al., 2004; Norsk, 2020). Total power spectrum and BRS also seems to decrease in space (Cooke et al., 1985; Iwasaki et al., 2004), probably due to baroreceptor overload and saturation, making it difficult for the ANS to modulate HR and blood pressure (Norsk et al., 2015). The accumulation of blood volume in the trunk also increases cerebrovascular pressure and engorgement of the neck vasculature, which stimulates aortic and carotid baroreceptors, affecting their saturation levels (Shen and Frishman, 2019). Thus, BRS decreases, probably, as a consequence of the regulation failure (Shen and Frishman, 2019).

The accumulation of blood in the neck region, and consequently increase of blood vessels pressure, is particularly worrisome for middle cerebral artery, CA and JV, once it involves impaired cerebral perfusion, stiffness of cerebral arteries and increased intracranial pressure. It is interesting to note that JV CSA have the same behavior in the three conditions, as shown in Figure 6. This indicates that the accumulation of blood in the neck is present both acutely and chronically, regardless of the microgravity model used. Some astronauts even presented JV stagnant or reversed venous flow (Marshall-Goebel et al., 2019). In this case, intracranial pressure is the main cause of Spaceflight Associated Neuro-Ocular Syndrome (SANS). More than half of astronauts exhibit at least one symptom of SANS, which symptoms are the same as patients with idiopathic intracranial hypertension (Norsk et al., 2015) and could cause permanent deficits in vision (Wojcik et al., 2020).

Another important issue is orthostatic intolerance (OI), a frequent occurrence resulting from reduced cardiac filling, inadequate SV, and/or compensatory neurohumoral responses (Goswami et al., 2017; Jordan et al., 2022). This leads to a failure in the maintenance of cerebral perfusion during postural alterations, such as transitions from sitting to standing or during more intensive physical activity (Mulavara et al., 2018; Jordan et al., 2022). As suggested by Neurolab and WISE-2005 studies (Arbeille et al., 1985; Dyson et al., 2007; Guinet et al., 2009), possible causes of OI include hypovolemia, increased vascular compliance, impaired arterial resistance and venous return, and cardiac atrophy (Goswami et al., 2017; Shen and Frishman, 2019; Jordan et al., 2022), very similarly to aging and immobility. Also, reduced BRS may contribute to OI (Jordan et al., 2022). Therefore, OI poses a relevant concern not only during emergency spacecraft landings, but also when returning to normogravity.

Also of interest, some biomarkers associated with the aging process (De Favari Signini et al., 2022), including mitochondrial dysfunction, deoxyribonucleic acid (DNA) damage, telomere shortening, and cellular senescence, have been observed in astronauts (Capri et al., 2023). One such study was the National Aeronautics and Space Administration (NASA) Twin Study (Garrett-Bakelman et al., 2019), which found that the astronaut twin had more noticeable aging indications compared to his ground-based twin, suggesting accelerated aging in the astronaut (Garrett-Bakelman et al., 2019). Interestingly, these biomarker changes, along with inflammation, arterial stiffness and significant risk of cardiovascular diseases, have been observed in both aging and spaceflight (Jaruchart et al., 2016; De Favari Signini et al., 2022; Capri et al., 2023; Hart, 2023; Malhan et al., 2023). Finally, it is noteworthy that the astronauts in the included articles were nearly 15 years older than the subjects in simulations. Thus, in addition to radiation exposure, sleep deprivation, and other stressors inherent to SF, the age difference may also have influenced the astronauts’ outcomes. Accordingly, the results of this review should be regarded with caution, and the findings of simulations cannot be fully extrapolated to astronauts.

There were several limitations in this review, including the small sample sizes used in the included studies, high variability in time exposure to microgravity or models/simulation, varied control groups, and the use of the same volunteers for different outcome measurements. Nonetheless, the challenge of conducting this kind of research justifies such limitations. Moreover, the systematic extraction and classification of outcomes, as well as the meta-analysis undertaken, produced robust and convincing evidence.

Future studies should consider the physical deconditioning of astronauts and its negative impact on their performance at work, in daily life in the spacecraft and especially during extravehicular exploratory activities. Therefore, understanding what happens to the physiology of the human body when exposed to microgravity is essential for monitoring and creating countermeasure strategies for the harmful mechanisms caused by fluid shift, supporting humanity to reach new planets.

## Conclusion

This review identified that weightlessness can negatively affect cardiac functions, vascular health, and cardiovascular autonomic modulation. These changes impact the health of astronauts during spaceflight and persist even after returning to Earth, resembling the aging process and prolonged immobility, potentially increasing the risk of cardiovascular diseases and other aging-related conditions. Further research is necessary to develop countermeasures against weightlessness to facilitate long space travels.

## Figures and Tables

**Table:** 

**AIx**	Augmentation index
**ANS**	Autonomic nervous system
**AS**	Acute Simulation
**BMI**	Body mass index
**BP**	Blood pressure
**BPV**	Blood pressure variability
**BRS**	Baroreflex sensitivity
**CA**	Carotid artery
**CAM**	Cardiovascular autonomic modulation
**Chi2**	Chi square test
**CI**	Confidence interval
**CO**	Cardiac output
**CO2**	Carbon dioxide
**CS**	Chronic Simulation
**CSA**	Cross-sectional area
**CVRR**	Coefficient of variation of RR intervals
**DAP**	Diastolic arterial pressure
**DF**	Degrees of freedom
**DI**	Dry Immersion
**DNA**	Deoxyribonucleic acid
**ECG**	Electrocardiography
**EF**	Ejection fraction
**FA**	Femoral artery
**FMD**	Flow-mediated dilation
**FTA**	Fraction time active
**G**	Gravity
**GastV**	Gastrocnemius vein
**HDT**	Head-Down Tilt
**HF**	High frequency
**HR**	Heart rate
**HRV**	Heart rate variability
**I2**	Heterogeneity measure
**IMT**	Intima-media thickness
**ISS**	International Space Station
**JV**	Jugular vein
**LA**	Left atrium
**LF**	Low frequency
**LF/HF ratio**	Ratio of low frequency and high frequency of heart rate variability
**LV**	Left ventricle
**LVDV**	Left ventricle diastolic volume
**LVET**	Left ventricular ejection time
**LVETi**	Left ventricular ejection time index
**LVSV**	Left ventricle systolic volume
**MAP**	Mean arterial pressure
**MCA**	Middle cerebral artery
**MCV**	Middle cerebral vein
**MD**	Mean difference
**MESOR**	Midline statistic of rhythm
**Mitral A**	Peak mitral flow during atrial systole
**Mitral E**	Peak mitral flow velocity during early filling
**Mitral E/A ratio**	Ratio between mitral E and A
**MRI**	Magnetic resonance imaging
**NASA**	National Aeronautics and Space Administration
**NN intervals**	Normalized time between two successive R-waves of the QRS signal on the electrocardiogram
**OI**	Orthostatic intolerance
**PCA**	Posterior cerebral artery
**PEP**	Pre-ejection period
**PEP/LVET**	Ratio between pre-ejection period and left ventricular ejection period
**PEPi**	Pre-ejection period index
**PF**	Parabolic Flight
**pNN50**	Percentage of adjacent NN (R-R) intervals that differ from each other by more than 50 ms
**PP**	Pulse pressure
**PV**	Portal vein
**PWTT**	Pulse wave transit time
**PWV**	Pulse wave velocity
**QRS-T angle**	Angle between the directions of ventricular depolarization and repolarization
**QS2**	Total electromechanical systole
**QS2i**	Total electromechanical systole index
**RA**	Right atrium
**RMSSD**	Root mean square of successive differences between normal heartbeats
**RR interval**	Time elapsed between two successive R-waves of the QRS signal on the electrocardiogram
**SANS**	Spaceflight Associated Neuro-Ocular Syndrome
**SAP**	Systolic arterial pressure
**SD**	Standard deviation
**SDARR**	Standard deviation of the set of averaged RR intervals
**SDNN**	Standard deviation of the interbeat intervals for all sinus beats
**SDRR**	Standard deviation of the R-R intervals
**SF**	Spaceflight
**SV**	Stroke volume
**SVR**	Systemic vascular resistance
**TibV**	Tibial vein
**ULV**	Ultra-low frequency
**VLF**	Very low frequency
**WI**	Head-out water immersion
**Z**	Z-score

## Data Availability

The original contributions presented in the study are included in the article/Supplementary Material, further inquiries can be directed to the corresponding authors.
