# Supplementary material for: Effects of weightlessness on the cardiovascular system: a systematic review and meta-analysis
Source: Front Physiol. 2024 Jul 26;15:1438089. doi: 10.3389/fphys.2024.1438089 (PMC11310543; doi:10.3389/fphys.2024.1438089)
Supplement: Supplementary file 1 [file Table1.pdf]

Table 1S. Databases and string.

Database	String	Filters applied
CINAHL	("Humans" OR "Astronauts") AND ("Weightlessness" OR "Weightlessness Simulation" OR "Space Flight") AND ("Cardiovascular System" OR "Hemodynamics" OR "Fluid Shifts")	Time Range: 2013 to 2023
Cochrane Library	("Humans" OR "Astronauts") AND ("Weightlessness" OR "Weightlessness Simulation" OR "Space Flight") AND ("Cardiovascular System" OR "Hemodynamics" OR "Fluid Shifts")	Time Range: 2013 to 2023
Scopus	("Humans" OR "Astronauts") AND ("Weightlessness" OR "Weightlessness Simulation" OR "Space Flight") AND ("Cardiovascular System" OR "Hemodynamics" OR "Fluid Shifts")	Research Articles; English; Time Range: 2013 to 2023.
Science Direct	("Humans" OR "Astronauts") AND ("Weightlessness" OR "Weightlessness Simulation" OR "Space Flight") AND ("Cardiovascular System" OR "Hemodynamics" OR "Fluid Shifts")	Research Articles; Time Range: 2013 to 2023.
PubMed	("Humans"[Mesh] OR "Astronauts"[Mesh]) AND ("Weightlessness"[Mesh] OR "Weightlessness Simulation"[Mesh] OR "Space Flight"[Mesh]) AND ("Cardiovascular System"[Mesh] OR "Hemodynamics"[Mesh] OR "Fluid Shifts"[Mesh])	Humans; English; Time Range: 2013 to 2023.
Web of Science	("Humans" OR "Astronauts") AND ("Weightlessness" OR "Weightlessness Simulation" OR "Space Flight") AND ("Cardiovascular System" OR "Hemodynamics" OR "Fluid Shifts")	Research Articles; English; Time Range: 2013 to 2023.

Searching databases using specific combinations for each database and all applied filters.
